# Novel donor-π-acceptor benzimidazole-based chromophores: synthesis, antitumor assessment, and pharmacokinetics

**DOI:** 10.1039/d6ra00254d

**Published:** 2026-03-11

**Authors:** Salhah D. Al-Qahtani, Ghadah M. Al-Senani, Khaled M. Elattar

**Affiliations:** a Department of Chemistry, College of Science, Princess Nourah bint Abdulrahman University P.O. Box 84428 Riyadh 11671 Saudi Arabia; b Unit of Genetic Engineering and Biotechnology, Mansoura University El-Gomhoria St. Mansoura 35516 Egypt khaledelattar2@mans.edu.eg

## Abstract

Targeted chromophores, 2-(*N*,*N*-dialkyl-/diaryl-aminophenyl)benzimidazole hybrids 2a, 2b, 4a, and 4b, were designed and synthesized. The designed hybrids were identified as donor-π-acceptor (D-π-A) structures, incorporating *N*,*N*-dialkyl/diarylamino groups as the donor moiety and nitro or pyridinyl Schiff-base groups as the acceptor moiety. The hybrids were found to exhibit significant solvent-dependent properties, especially in polar solvents such as DMSO, which enhanced their fluorescence properties. Conjugate 2a exhibited absorbance and fluorescence maxima at 390 and 528 nm, respectively, in DMSO, while hybrid 2b demonstrated absorbance and fluorescence maxima at 396 and 536 nm, respectively. Hybrids 4a and 4b exhibited similar solvent-dependent activity. The cytotoxicity of all the synthesized chromophores was tested against several human cancer cell lines using an MTT assay, and their inhibitory activity against VEGFR-2 was determined using an anti-phosphotyrosine-based quantitative kinase assay. Chromophore 4a exhibited the highest overall cytotoxicity, with IC_50_ = 12.64 ± 0.29 µM towards HepG2 cells and IC_50_ = 12.19 ± 0.30 µM towards PC3 cells. Meanwhile, the four targeted chromophores were tested towards VEGFR-2 using the reference sorafenib, which is a noticeable target in anti-angiogenic cancer therapy. The docking results revealed that hybrid 4b exhibited the strongest binding score, which was close to that of sorafenib (reference), suggesting that it shows the most promising profile. Analysis by SwissADME revealed that hybrids 2a and 4a exhibited good pharmacokinetic properties, such as high gastrointestinal absorption and blood–brain barrier permeability. In conclusion, the combined experimental and computational approach presented here suggests the potential of these benzimidazole chromophores as early leads for anticancer and anti-angiogenic agents.

## Abstract 2

Benzimidazole hybrids showed selective cytotoxicity against HepG2, PC3, and MCF-7 cancer cells with low toxicity to WI-38 cells. VEGFR-2 inhibition and docking confirmed strong binding, indicating promising anticancer and anti-angiogenic activity.

## Graphical abstract

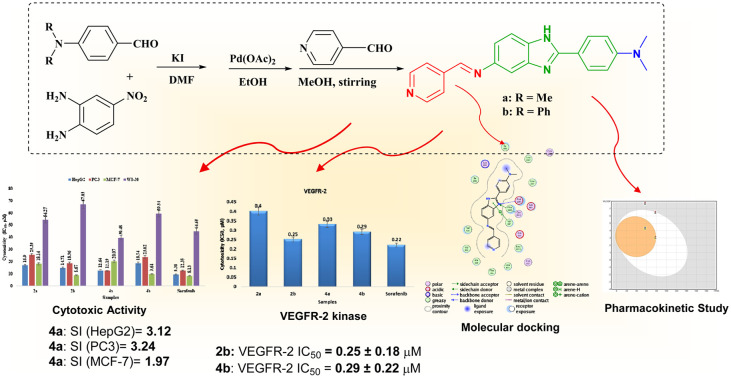

## 1. Introduction

The design and synthesis of new fluorescent chromophores have attracted a lot of research attention because of their numerous uses in biological imaging, optoelectronic systems, and photodynamic treatment.^1–3^ However, donor-π-acceptor (D-π-A) skeletons are particularly attractive because of their variable photophysical structures.^4^ In them, the presence of strong electron-donating and electron-withdrawing moieties connected by a π-conjugated link advances intramolecular charge transfer (ICT), resulting in pronounced fluorescence features.^5–7^ Benzimidazole, a fused heterocyclic moiety formed by benzene and imidazole rings, is a valuable building block in the expansion of these systems because of its intrinsic fluorophores and chemosensors, stability, and biological activity.^8–10^ Recent research has focused on benzimidazole hybrids and their potential anticancer applications.^11^ Their ability to interact with biological targets, including DNA strands and proteins, *via* electron-π stacking, hydrogen interactions, and van der Waals bonds, makes them promising candidates for drug development.^12^

Meanwhile, the insertion of diverse functional groups into the benzimidazole core may enhance its inhibitory effect on the growth of many types of cancer cells.^13–15^ Consequently, the synthesis of new benzimidazole-based D-π-A fluorescent chromophores is imperative.^16^ These conjugations are engineered to exhibit high antiproliferative activity and fluorescence, enabling the monitoring of intracellular distribution. Electron-donating moieties are typically derivatives of amino, hydroxyl, and methoxy groups.^17^ Nevertheless, electron-withdrawing groups, such as nitro, cyano, and other carbonyl groups, pull the bond electrons.^18^ The π-bridge frequently involves conjugated systems, such as phenyl, thiophene, or vinyl bridges, which enable effective charge transfer and fluorescence emission.^19,20^

**Fig. 1:**
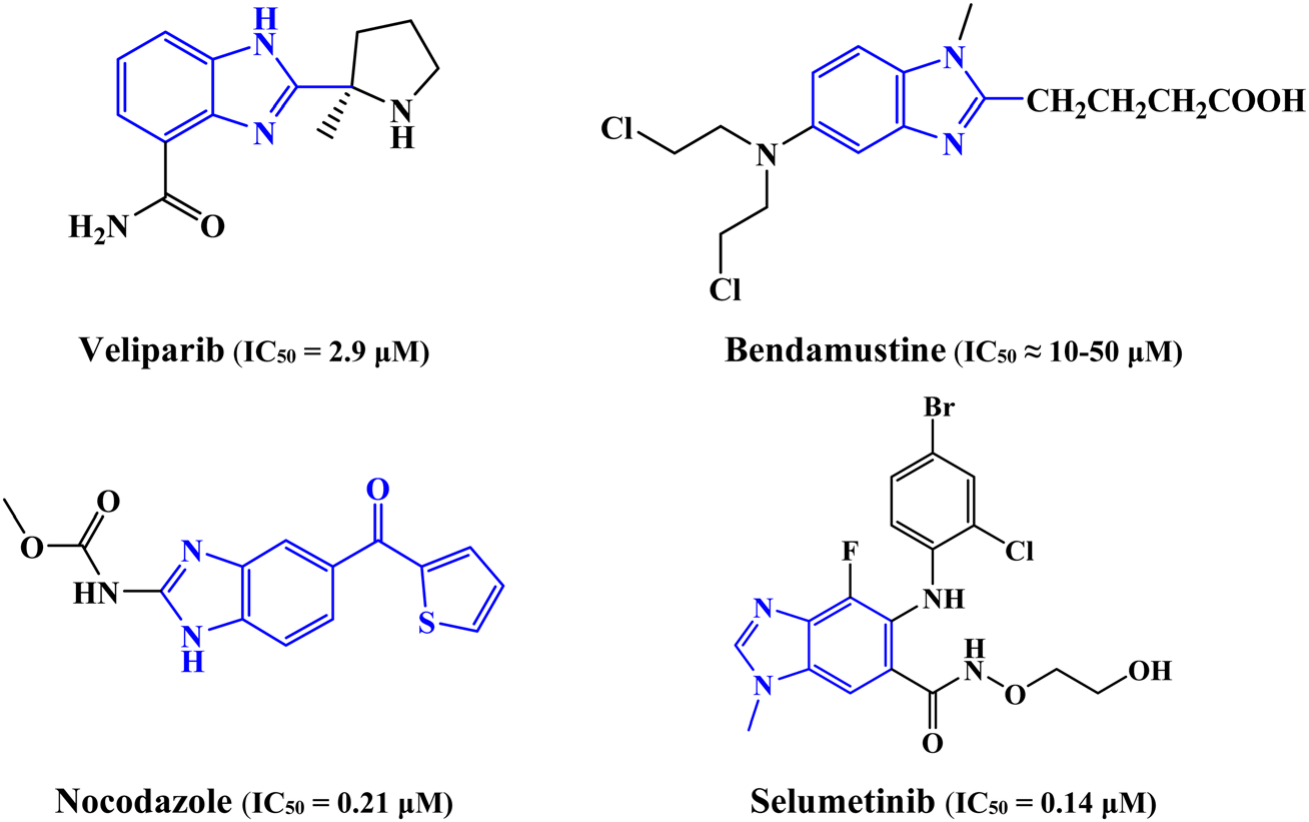
Benzimidazole-containing anticancer drugs.

In addition to their biological activities, the evaluation of their pharmacokinetic profile is important for the early assessment of the drug-like characteristics of newly synthesized compounds^21–23^ (Fig. 1). Pharmacokinetics refers to the absorption, distribution, metabolism, and excretion (ADME) of substances.^24–26^ Moreover, theoretical molecular docking studies are critical for understanding the bindings of the original drugs with target proteins across their amino acids.^27^ By modeling the docking process, researchers have gained a better understanding of how chromophores bind, align, and interact with biomolecule active sites. This information is crucial for understanding their antiproliferative effect and tailoring their structure for maximum effectiveness. These tests provide information on the effectiveness of chromophores and their potential as chemotherapeutic drugs.

With these aspects in mind, herein, we describe the rational design, synthesis, and comprehensive evaluation of a new family of benzimidazole-derived D-π-A chromophores (2a, 2b, 4a, and 4b). The photophysical properties of these compounds were first examined in various solvents with different polarities. This was followed by a comprehensive evaluation of their cytotoxicity against a variety of cancer cell lines and VEGFR-2 inhibition as a potential anti-angiogenic agent using sorafenib as a positive control. Molecular docking and SwissADME studies were conducted to interpret the results and predict the ADME properties. In summary, this work is aimed at finding new benzimidazole hybrids with potential fluorescent, anticancer, and drug-like properties.

## 2. Experimental

### 2.1. Materials and methods

The melting points of the prepared hybrids were defined using a Gallenkamp melting point apparatus and are uncorrected. IR spectra (potassium bromide) were recorded using a Thermo Scientific iS10 FTIR spectrometer. Meanwhile, ^1^H and ^13^C NMR spectra were acquired using a JEOL spectrometer operating at 500 MHz with DMSO-*d*_6_ as the solvent; the chemical shifts are reported in ppm relative to TMS. Mass spectra were recorded on a DSQII GC-MS instrument operating at 70 eV. UV-vis spectra were measured using a Shimadzu UV-3600 spectrophotometer, while fluorescence emission spectra were obtained using a Horiba FS5 spectrofluorometer. Elemental (C, H, N) analyses were performed using a PerkinElmer 2400 elemental analyzer, and the results were within ±0.4% of the calculated values.

### 2.2. Synthesis of the *N*,*N*-dimethyl- or *N*,*N*-diphenyl-aminophenyl,5-nitrobenzimidazole hybrids 2a and 2b

4-Nitro-*o*-phenylenediamine (1.64 g, 12 mmol) was dissolved in 30 mL of DMF. Then, 4-formyl-*N*,*N*-dimethylanilin 1a or 4-formyl-*N*,*N*-diphenylanilin 1b (12 mmol) and KI (0.2 g, 1.2 mmol) were added to the mix. The solution was subjected to reflux at 150 °C for 12 h. The solution was left to cool and diluted by adding 500 mL of cold water. The obtained solid was purified and then cleaned by recrystallization from a combination of ethanol and dimethylformamide (2 : 1 ratio). This process yielded the desired *N*,*N*-dimethyl- or *N*,*N*-diphenyl-aminophenyl,5-nitrobenzimidazole hybrids, namely, 2a and 2b, respectively.

#### 2.2.1. *N*,*N*-dimethyl-4-(5-nitro-1*H*-benzimidazolyl)aniline (2a)

Yield = 77%, m.p. = 216 °C-217 °C. IR (*ν*/cm^−1^): 3051, 2952, 1651, 1524, 1476, 1352, 1173. ^1^H NMR (*δ*/ppm): 2.88 (s, 6H, 2CH_3_), 6.82 (d, *J* = 8.99 Hz, 2H, Ar–H), 7.71 (d, *J* = 8.99 Hz, 2H, Ar–H), 8.23 (d, *J =* 8.49 Hz, 1H, benzimidazolyl-H_7_), 8.34 (dd, *J =* 8.49 Hz, 1H, benzimidazolyl-H_6_), 9.11 (d, *J =* 2.50 Hz, 1H, benzimidazolyl-H_4_), 11.14 (s,1H, benzimidazolyl-H_1_). ^13^C NMR (*δ*/ppm): 42.54 (2C), 114.11 (2C), 118.23, 119.44, 122.42, 128.93 (2C), 140.28, 145.80, 153.58, 155.70, 183.36. MS *m*/*z* (%): 282 (M^+^, 67.66). Analysis of C_15_H_14_N_4_O_2_ (282.30): calculated: C, 63.82; H, 5.00; N, 19.85%. Found: C, 63.80; H, 5.97; N, 19.82%.

#### 2.2.2. 4-(5-Nitrobenzimidazolyl)-*N*,*N*-diphenylaniline (2b)

Yield = 68%, m.p. = 283 °C-284 °C. IR (*ν*/cm^−1^): 3375, 33.7, 1628, 1539, 1839, 1358, 1262. ^1^H NMR (*δ*/ppm): 6.99 (d, *J* = 8.00 Hz, Ar–H), 7.16–7.44 (m, 10H, Ar–H), 7.77 (d, *J* = 8.00 Hz, 2H, Ar–H), 8.20 (d, *J =* 8.40 Hz, 1H, benzimidazolyl-H_7_), 8.36 (dd, *J =* 8.40 Hz, 1H, benzimidazolyl-H_6_), 9.23 (d, *J =* 2.40 Hz, benzothiazolyl-H_4_), 11.70 (s,1H, benzimidazolyl-H1). ^13^C NMR (*δ*/ppm): 118.39, 119.45, 122.39, 125.49 (2C), 126.28 (4C), 127.29, 129.47 (2C), 129.86 (4C), 131.58 (2C), 141.75, 145.86, 146.26 (2C), 150.13, 154.42, 168.12. MS *m*/*z* (%): 406 (M^+^, 59.43). Analysis of C_25_H_18_N_4_O_2_ (406.45): calculated: C, 73.88; H, 4.46; N, 13.78%. Found: C, 73.79; H, 4.58; N, 9.67%.

### 2.3. Synthesis of the 5-amino-2-(*N*,*N*-dimethyl- or *N*,*N*-diphenyl-aminophenyl)-benzimidazole hybrids 3a and 3b

Aqueous hydrazine hydrate (31 mL) and Pd(CH_3_COO)_2_ (40 mg, 1.6 mmol) were added to a solution of *N*,*N*-dimethyl,5-nitrobenzimidazolylaniline (2a) or 5-nitrobenzimidazolyl,*N*,*N*-diphenylaniline (2b) (5.5 mmol) in 50 mL of ethyl alcohol. The combination was refluxed for one hour and then filtered. The obtained product was diluted with an ethanol/water mixture and treated dropwise with a dilute aqueous solution of Na_2_S with stirring at room temperature. The mixture was then filtered to remove the black PbS precipitate, and the filtrate was thoroughly washed with water. The organic phase was subsequently concentrated, and the crude product was purified by recrystallization. The gray solids of the conformist 5-aminobenzimidazole hybrid 3a or 3b were produced by crystallizing ethanol.

#### 2.3.1. 2-(*N*,*N*-dimethylamino)phenyl-1*H*-benzimidazol-5-amine (3a)

Yield = 79%, m.p. = 189 °C. IR (*ν*/cm^−1^): 3467, 3311, 2917, 1623, 1539, 1239, 1163. ^1^H NMR (*δ*/ppm): 2.89 (s, 6H, –N(CH_3_)_2_), 5.28 (s, 2H, NH_2_), 6.64 (dd, *J =* 8.40 Hz, 1H, benzimidazolyl-H_6_), 6.72 (d, *J =* 2.40 Hz, 1H, benzimidazolyl-H_4_), 6.91 (d, *J* = 8.00 Hz, Ar–H), 7.47 (d, *J =* 8.40 Hz, 1H, benzimidazolyl-H_7_), 7.81 (d, *J* = 8.00 Hz, 2H, Ar–H), 11.33 (s,1H, benzimidazolyl-H1). ^13^C NMR (*δ*/ppm): 42.47 (2C), 104.92, 114.32 (2C), 116.88, 120.35, 123.67, 126.12, 129.80 (2C), 148.33, 154.61, 156.15, 167.77. MS *m/z* (%): 252 (M^+^, 73.24). Analysis of C_15_H_16_N_4_ (252.32): calculated: C, 71.38; H, 6.38; N, 22.20%. Found: C, 71.35; H, 6.43; N, 22.18%.

#### 2.3.2. 2-(4-(*N*,*N*-diphenylamino)phenyl)benzimidazol-5-amine (3b)

Yield = 74%, m.p. = 185 °C. IR (*ν*/cm^−1^): 3453, 3365, 3147, 1647, 1549, 1452, 1420, 1255. ^1^H NMR (*δ*/ppm): 6.09 (s, 2H, NH_2_), 6.61 (dd, *J =* 8.40 Hz, 1H, benzimidazolyl-H_6_), 6.80 (d, *J =* 2.50 Hz, 1H, benzimidazolyl-H_4_), 6.92 (d, *J* = 8.40 Hz, 2H, Ar–H), 7.10–7.44 (m, 10H, Ar–H), 7.63 (d, *J =* 8.40 Hz, 1H, benzimidazolyl-H_7_), 7.82 (d, *J* = 8.40 Hz, 2H, Ar–H), 12.43 (s,1H, benzimidazolyl-H_1_). ^13^C NMR (*δ*/ppm): 106.57, 115.17, 121.86, 122.71, 125.41 (2C), 126.62 (4C), 127.50, 128.49 (2C), 130.44 (4C), 132.07 (2C), 145.39 (2C), 148.21, 150.22, 154.34, 167.59. MS *m/z* (%): 376 (M^+^, 58.28). Analysis of C_25_H_20_N_4_ (376.46): calculated: C, 79.76; H, 5.36; N, 14.88%. Found: C, 76.69; H, 5.43; N, 10.93%.

### 2.4. Synthesis of the *N*,*N*-dimethyl- or *N*,*N*-diphenyl-(5-((pyridin-4-ylmethylene)amino)-benzimidazol-2-yl)anilinehybrids 4a and 4b

Isonicotinaldehyde (0.11 g, 1 mmol) was dissolved in 12 mL of methyl alcohol and added dropwise to an aqueous solution of 5-aminobenzimidazole derivatives 3a or 3b (1 mmol) dissolved in 12 mL of methyl alcohol. The admixed suspension was disturbed overnight. The resulting mixture was purified yielding the yellow product of the benzimidazole-pyridine hybrids 4a and 4b, respectively. The lead content in the purified compounds 3a, 3b, 4a, and 4b was analyzed by ICP-MS and found to be below the LOQ (0.1 ppm), ensuring compliance with ICH Q3D(R1) guidelines.

#### 2.4.1. *N*,*N*-dimethyl(5-((pyridinylmethylene)amino)-1*H*-benzimidazolyl)aniline (4a)

Yield = 76%, m.p. = 293 °C-294 °C. IR (*ν*/cm^−1^): 3320, 3208, 2925, 1643, 1618, 1559, 1237. ^1^H NMR (*δ*/ppm): 3.11 (s, 6H, 2CH_3_), 6.83 (d, *J* = 8.40 Hz, 2H, Ar–H), 7.26 (dd, *J =* 8.40 Hz, 1H, benzimidazolyl-H_6_), 7.68 (d, *J* = 8.40 Hz, 2H, Ar–H), 7.79 (d, *J =* 8.40 Hz, 1H, benzimidazolyl-H_7_), 7.93 (d, *J =* 4.40 Hz, 2H, pyridyl-H_3_, H_5_), 8.14 (d, *J =* 2.50 Hz, 1H, benzothiazolyl-H_4_), 8.73 (d, *J =* 4.40 Hz, 2H, pyridyl-H_2_, H_6_), 8.84 (s, 1H, CH = N), 11.63 (s,1H, benzimidazolyl-H1). ^13^C NMR (*δ*/ppm): 41.46 (2C), 114.01 (2C), 117.86, 120.42 (2C), 121.19, 121.72, 123.62, 130.03 (2C), 135.01, 142.43, 147.84, 150.98 (2C), 154.71, 156.67, 160.93, 168.33. MS *m*/*z* (%): 341 (M^+^, 64.52). Analysis of C_21_H_19_N_5_ (341.42): calculated: C, 73.88; H, 5.61; N, 20.51%. Found: C, 73.79; H, 5.68; N, 20.46%.

#### 2.4.2. *N*,*N*-diphenyl(5-((pyridinylmethylene)amino)-1*H*-benzoimidazolyl)aniline(4b)

Yield = 69%, m.p. = 279 °C-280 °C. IR (*ν*/cm^−1^): 3352, 3063, 1648, 1549, 1256. ^1^H NMR (*δ*/ppm): 6.83 (d, *J* = 8.40 Hz, 2H, Ar–H), 7.14–7.22 (m, 6H, Ar–H), 7.30 (dd, *J =* 8.40 Hz, 1H, benzimidazolyl-H_6_), 7.42–7.44 (m, 4H, Ar–H), 7.66 (d, *J* = 8.40 Hz, 2H, Ar–H), 7.84 (d, *J =* 8.40 Hz, 1H, benzimidazolyl-H_7_), 7.93 (d, *J =* 4.40 Hz, 2H, pyridyl-H_3_, H_5_), 8.46 (d, *J =* 2.50 Hz, 1H, benzimidazolyl-H_4_), 8.82 (d, *J =* 4.40 Hz, 2H, pyridyl-H_2_, H_6_), 8.93 (s, 1H, CH = N), 11.55 (s,1H, benzimidazolyl-H1). ^13^C NMR (*δ*/ppm): 112.63, 116.45 (2C), 119.18, 121.35, 122.09 (2C), 123.64 (4C), 126.42, 127.19 (2C), 128.37 (4C), 129.80 (2C), 130.12, 145.20, 147.98 (2C), 148.01, 150.48, 151.22 (2C), 155.78, 161.44, 173.70. MS *m/z* (%): 465 (M^+^, 49.61). Analysis of C_31_H_23_N_5_ (456.02): calculated: C, 79.98; H, 4.98; N, 15.04%. Found: C, 80.01; H, 4.94; N, 15.11%.

### 2.5. *In vitro* cytotoxicity evaluation

The *in vitro* cytotoxic effectiveness of the newly synthesized benzimidazole hybrids 2a, 2b, 4a, and 4b was evaluated using the MTT assay against human breast adenocarcinoma (MCF-7), prostatic adenocarcinoma (PC3), hepatoblastoma (HepG2), and normal human fetal lung fibroblast (WI-38) cell lines. All biological assays were performed at the National Research Centre, Giza, Egypt, from which the authenticated cell lines were obtained and maintained under standard sterile culture conditions.^28–30^ All cell culture reagents, including RPMI-1640 medium, fetal bovine serum, penicillin, and streptomycin, were obtained from Thermo Fisher Scientific and used as received.

To obtain the dose–response curves for the cytotoxicity of the assay and VEGFR-2 inhibitory activity, at least five different concentrations of the compounds, ranging from 0.1 × to 10 × IC_50_, were assessed and evaluated by the data-fitting analysis of the four-parameter logistic equation. All data points were obtained from experiments carried out in triplicate and are expressed as the mean ± SD. A significant sigmoidal curve and a consistent Hill slope and *R*^2^ value are indicative of the concentration dependency of the biological activities of the compounds. Graphs depicting the dose–response curve and the associated statistical parameters for the compounds are provided in Fig. S1 and Table S1, respectively.

### 2.6. *In vitro* VEGFR-2 kinase inhibition

The VEGFR-2 kinase inhibition assay was performed at the National Research Centre, Giza, Egypt, using an enzyme-linked immunosorbent assay kit (Boehringer Mannheim, SA; currently Roche Diagnostics), according to the manufacturer's instructions.^31^ The mouse IgG anti-phosphotyrosine primary antibody and HRP-linked sheep anti-mouse immunoglobulin secondary antibody were supplied with the assay kit and used according to the manufacturer's instructions.

Dose–response curves were generated using at least five concentrations spanning from approximately 0.1 times to 10 times the IC_50_ value for each compound. All experiments were performed in triplicate, and data are presented as mean ± SD. Non-linear regression analysis was conducted using a four-parameter logistic (4 PL) model with a variable Hill slope, and the goodness of fit was assessed based on the sigmoidal curve shape, *R*^2^ values, and Hill slope consistency. The resulting VEGFR-2 inhibition profiles and associated statistical parameters are provided in Fig. S2 and Table S2 (SI).

### 2.7. Molecular modeling

In this study, all target hybrids were docked to understand how they bind to the VEGFR-kinase enzyme. We performed their docking processes using the MOE 2019 online version tool, which creates a distinctive binding between the protein and ligand. Through the utilization of 2OH4, we were able to systematically study the binding interactions of the synthesized benzimidazole hybrids (2a, 2b, 4a, and 4b) with key active-site residues, such as Glu883, Val897, Asp1044, and Cys1043, which are crucial for VEGFR-2 activity. Additionally, docking studies on this receptor have allowed a direct comparison to be made with sorafenib, a clinically used VEGFR-2 inhibitor. Both hybrids 2a-4b and the utilized protein were saved and deposited as MOL and PDB format files, respectively. The protein representative of the target VEGFR-kinase was obtained from the online RCSB (PDB:2OH4).^32^ To make sure the binding data was correct, we used sorafenib, a VEGFR-kinase inhibitor, as a standard ligand and compared the expected binding shapes to the real investigational results.^33^ This selection is important to ensure that the docking results are biologically relevant, reproducible, and informative, especially in the design of benzimidazole-based VEGFR-2 inhibitors.

### 2.8. Pharmacokinetic properties

We submitted the synthesized benzimidazoles to the online version of the SwissADME software using the Swiss Bioinformatics Institute (https://www.swissadme.ch/) to ascertain their drug-likeness, pharmacokinetic characteristics, and overall bioavailability. We investigated key parameters for instances, molecular weight (M. wt), lipophilicity (iLog *P*), topological polar surface area (TPSA), H-bond donors (HBD), H-bond acceptors (HBA), rotatable bonds (RT), Lipinski's rule of five, bioavailability scores, gastrointestinal absorption (GI), P-glycoprotein (Pgp) substrate status, and blood–brain barrier (BBB) permeability (Table S3).

### 2.9. Statistical analysis

All *in vitro* cytotoxicity and VEGFR-2 inhibition assays were carried out in triplicate wells for each concentration, and each experiment was conducted independently three times (*n* = 3). Results are expressed as mean ± standard deviation (SD). Statistical analyses were carried out using one-way ANOVA with Dunnett's test for multiple comparisons to the control group, and an unpaired *t*-test for comparison with sorafenib as appropriate. Exact *p*-values are provided in the figure legends and tables.

## 3. Results and discussion

### 3.1. Synthesis of the 2-(*N*,*N*-dimethylaminophenyl)-substituted-benzimidazole and 2-(*N*,*N*-diphenylaminophenyl)-substituted-benzimidazole hybrids

**Scheme 1:**
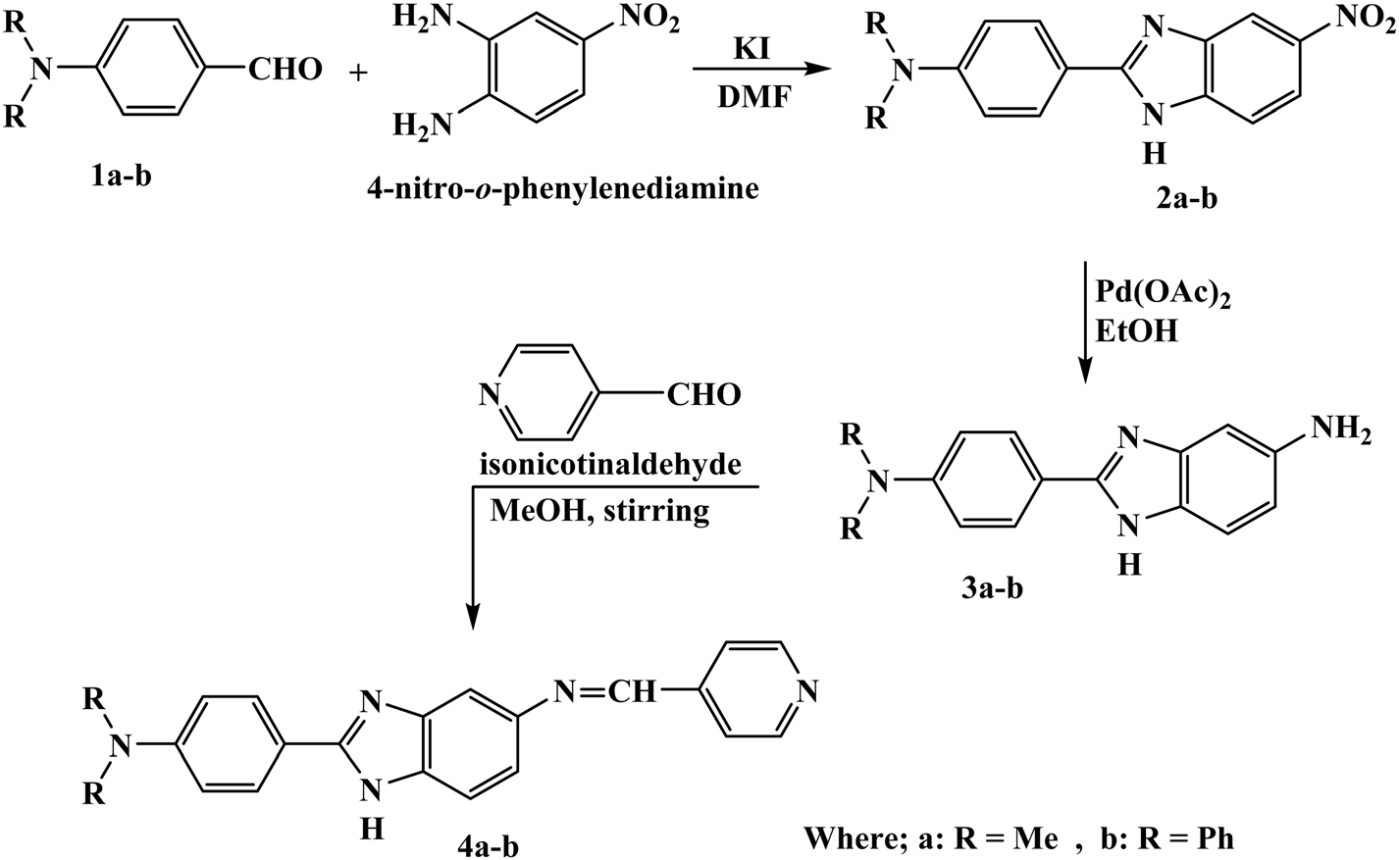
General synthetic route for the preparation of the *N*,*N*-dimethyl- and *N*,*N*-diphenyl-aminophenyl-substituted benzimidazole hybrids 2a, 2b, 3a, 3b, and their Schiff base derivatives 4a and 4b.

The synthesis method in this work enabled the preparation of the desired benzimidazole derivatives in moderate to good yields (68–77%), reflecting the efficiency of the condensation and reduction steps. Scheme 1 illustrates the synthetic directions for the benzimidazole hybrids 2a, 2b, 4a, and 4b. 4-Formyl-*N*,*N*-dimethylanilin (1a) and 4-formyl-*N*,*N*-diphenylanilin (1b) were refluxed separately with 4-nitro-*o*-phenylenediamine in the presence of a small amount of potassium iodide (catalyst) in DMF to afford the equivalent yield of 2-(*N*,*N*-dimethyl- or diphenyl-aminophenyl)-5-nitro-benzimidazole hybrids 2a and 2b, respectively.

**Scheme 2:**
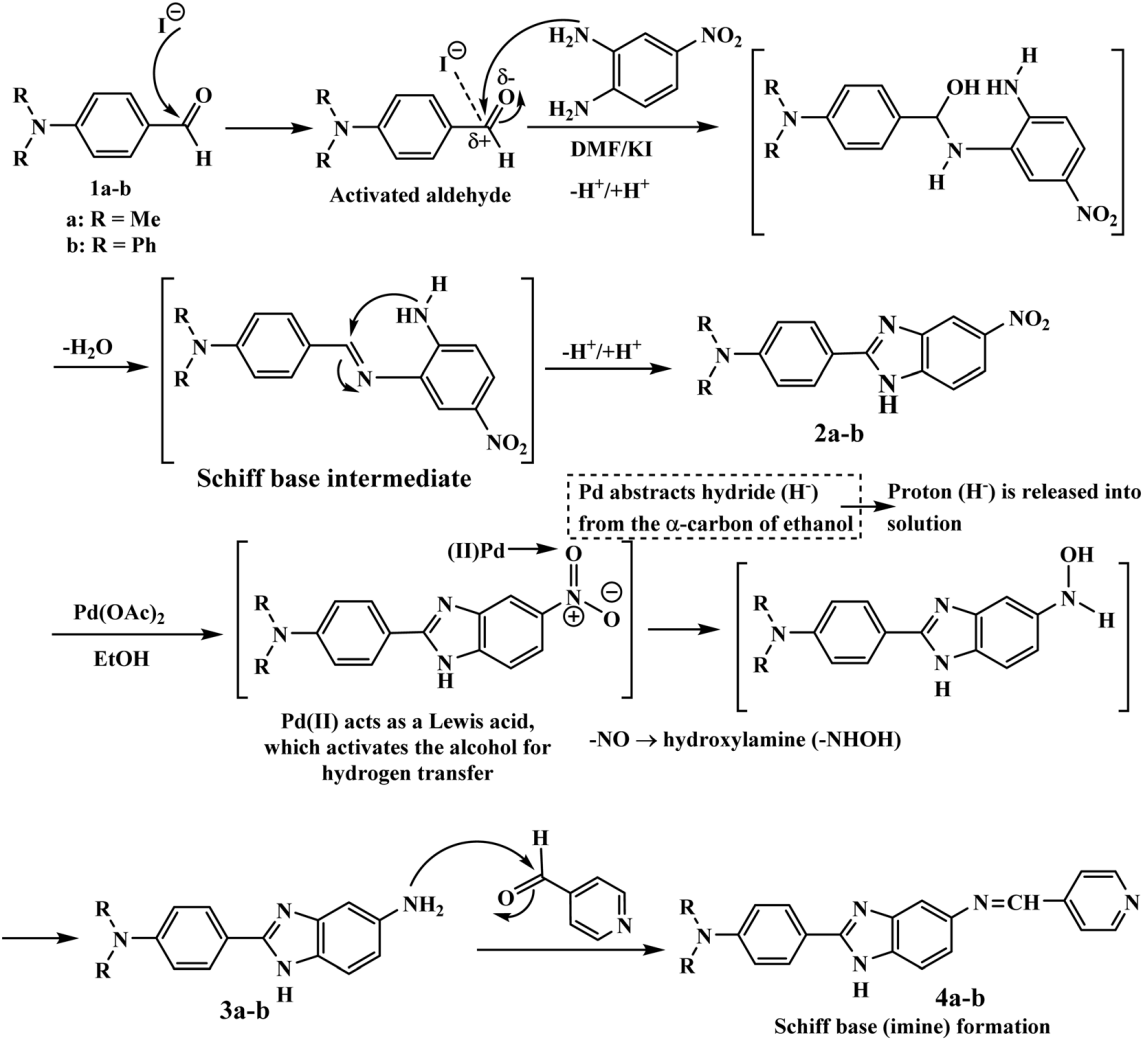
Proposed mechanism for the formation of the benzimidazole hybrids.

Potassium iodide efficiently catalyzed the condensation of 1a and 1b with 4-nitro-*o*-phenylenediamine in DMF (Scheme 2). Potassium iodide activates the aldehyde through polarization that offers an active nucleophilic attack and imine and/or Schiff base formation, followed by intramolecular cyclization and dehydration to afford 5-nitrobenzimidazole intermediates 2a and 2b.^34–36^ The nitro groups in 2a and 2b were selectively reduced into amino groups using palladium acetate in ethanol. The palladium catalyst enables hydrogen transfer from the solvent to afford 5-aminobenzimidazoles 3a and 3b while preserving the benzimidazole core. Finally, the condensation of 3a and 3b with isonicotinaldehyde in methanol affords an imine, followed by its dehydration to realize Schiff base hybrids 4a and 4b with precise functionalization at the 5-position of the benzimidazole ring.

The nitro groups in chromophores 2a and 2b were confirmed by their characteristic bands at 1524 and 1539 cm^−1^, respectively. The chemical structures of the synthesized benzimidazole conjugates 2a, 2b, 3a, 3b, 4a, and 4b were confirmed by FTIR, ^1^H-NMR, ^13^C-NMR, and mass spectrometry (Fig. S4–S6, S8–S10, S12–S14, S16–S18, S20–S22, and S24–S26, respectively). However, the crucial role of the potassium iodide catalyst in the condensation reaction for the formation of the benzimidazole chromophores 2a and 2b can be efficient in terms of the ability of the catalyst to activate the aldehyde functionality. The role of the iodide ion, in the polar aprotic solvent DMF, is to act as a strong nucleophile in the activation of the aldehyde group, thereby increasing the electrophilicity of the aldehyde group, which is attacked by the amino groups of *o*-phenylenediamine, thereby increasing the rate of formation of the imine (Schiff base) intermediate. So, the formation of the imine intermediate led to the intramolecular cyclization reaction, thereby leading to the formation of the benzimidazole nucleus. Moreover, the presence of the iodide ion increases the rate of the proton transfer reaction, thereby increasing the rate of the reaction through the condensation pathway under mild reaction conditions. Furthermore, the selective reduction of the nitro group to the corresponding 5-amino derivatives 3a and 3b was achieved using hydrazine hydrate in the presence of a catalytic amount of Pd(ii). The ongoing synthesis plan is aimed at obtaining 5-aminobenzimidazole hybrids 3a and 3b individually, by reducing 5-nitrobenzimidazole hybrids 2a and 2b through treatment with hydrazine hydrate in the presence of catalytic Pd(CH_3_COO)_2_. The formation of derivatives 3a and 3b was confirmed by their IR values at 1539 and 1549 cm^−1^ and in ^1^H-NMR spectra by signals at 5.28 and 6.09 ppm, respectively (Fig. S11–S18). Subsequent condensation of isonicotinaldehyde with 5-amino-2-benzimidazole hybrids 3a and 3b afforded the Schiff base hybrids 4a and 4b, whose structures were confirmed by IR, NMR, and mass analysis (Fig. S19–S26).

The variations in the yields of the compounds bearing dimethylamino and diphenylamino groups reveal the reaction efficiency factors in relation to steric and electronic environments. The addition of dialkyl-, as opposed to diaryl-amino groups, is likely to help control the electron-donating ability of the hybrids; this can influence the photophysical characteristics, including the fluorescent emission and the cytotoxicity of the hybrids. The overall synthesis procedure shows the ease and efficiency in synthesis, mild reaction conditions, and ease of purification, which are quite beneficial for carrying out further biological and photophysical studies of the hybrids.

### 3.2. Photophysical properties

**Fig. 2:**
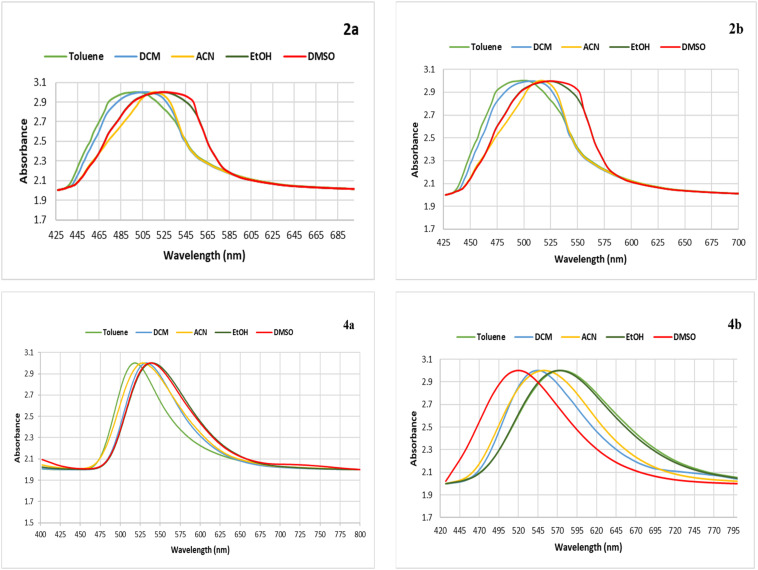
Fluorescence spectra of the benzimidazole derivatives 2a, 2b, 4a, and 4b in DMSO.

**Fig. 3:**
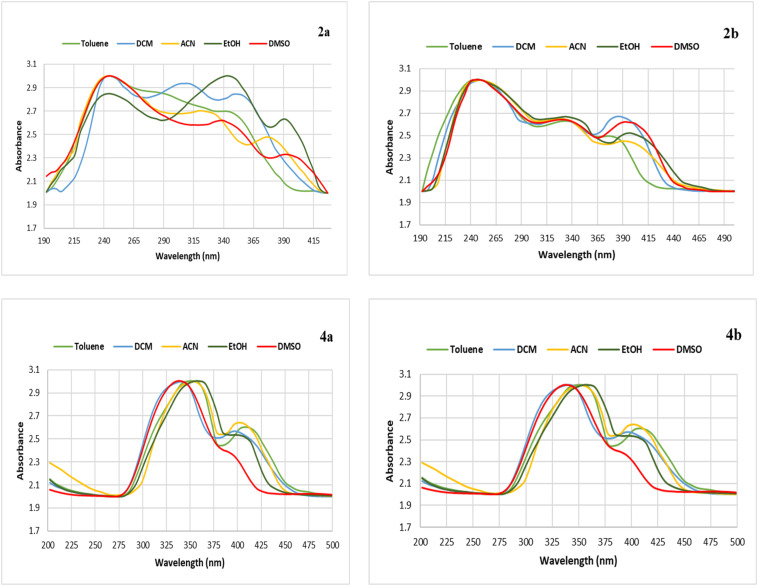
Absorption spectra of the benzimidazole derivatives 2a, 2b, 4a, and 4b in DMSO.

To investigate the photophysical properties of the synthesized chromophores, the absorption maxima were recorded in various solvents. The fluorescence behaviors of these chromophores are noticed in polar solvents, such as dimethyl sulfoxide (DMSO), which shows that the polarity of the solvent had a proper effect on their excited-state properties. Fluctuations in the absorbance maxima for toluene, dichloromethane, acetonitrile, and ethanol, all of which are polar solvents, signify that electronic interactions and conjugation are shifting. This is predicted by the idea that photophysical properties are heavily influenced by molecular structure and solvent interactions. For example, conjugate 2a has an absorbance maximum value at 390 nm and a fluorescence maximum value = 528 nm in DMSO, and conjugate 2b exhibits absorbance and fluorescence maxima at 396 nm and 536 nm, respectively, in the same solvent (Fig. 2 and 3).

**Fig. 4:**
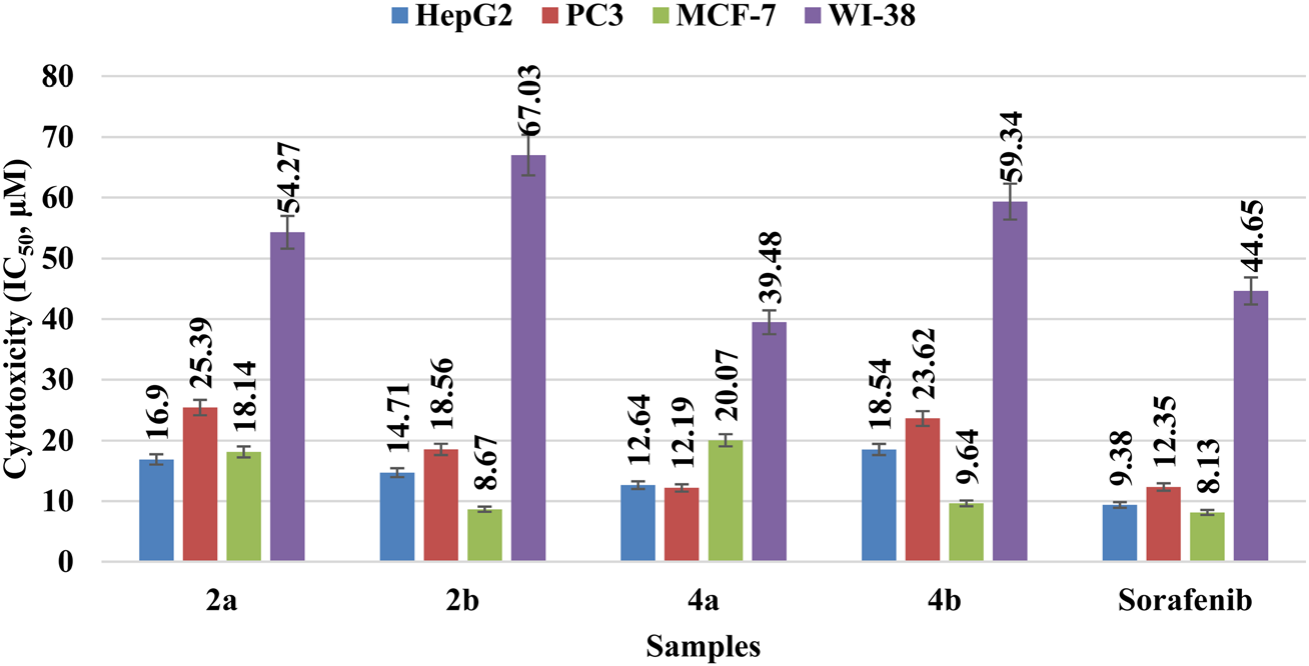
Cytotoxic efficacy . of the synthesized benzimidazole hybrids 2a, 2b, 4a, and 4b in terms of their *in vitro* cytotoxic activity against HepG2, PC3, and MCF-7 cancer cells, with WI-38 fibroblasts as a control (non-cancerous cells). Data represent the mean ± SD of three independent experiments. The significance of the differences was determined by one-way ANOVA coupled with Dunnett's test against sorafenib (<0.05).

The photophysical features of hybrids 4a and 4b are solvent-dependent to a similar extent (Table S1). These changes are most likely due to differences in solute–solvent interactions, which alter the electrical environment of the hybrids. Additionally, the changes in the UV-visible spectrum showing a bathochromic shift with increasing emission intensity for the benzimidazole derivatives in polar solvents can be accounted for by an intramolecular charge transfer process that takes place between the amino moieties as electron donors and the benzimidazole moiety as an electron acceptor. This assertion is supported by the differences in the fluorescence quantum yields (*φ*_f_) of compounds 2a and 4a, with *φ*_f_ values higher than those of 2b and 4b bearing *N*,*N*-diphenylamino groups. However, this may be dependent on minimal steric congestion and maximal π-conjugation in the dialkylated compounds. In 4a and 4b, the introduction of the Schiff base moiety further enhances the π-conjugation, leading to the redshifted absorption and emission bands compared with the corresponding 2a and 2b. These findings evidence the significant role of the character of the amino substituent and the π-extension through the Schiff base moiety in the control of the corresponding image processes. The polarity of the solvent and the efficient solvation effect may stabilize the image levels. These findings collectively illustrate the effectiveness of the donor-π-acceptor framework to modulate the fluorescent properties of the designed benzimidazole-based conjugates. Although the UV and fluorescence spectra of chromophores 2a-b, 3a-b, and 4a-b in DMSO (Fig. 4) indicate the role of the structural moieties on the electronic transitions of the benzimidazole moiety, chromophore 2 with the nitro group shows characteristic bands of a donor-π-acceptor (D-π-A) system. The strong electron-withdrawing nitro branch increases the LUMO energy level, making the intramolecular charge transfer (ICT) possible, which results in a red shift and an enhancement of the fluorescence intensity. With the reduction of the nitro group to the amino group in chromophore 3, the electronic character of the group changes from electron-withdrawing to electron-donating.

The balance of the push–pull system of the compound is disturbed, which further influences the ICT process. This is evident from the changes in the UV-vis and emission spectra. The amino group enhances the electron density of the benzimidazole core. It is also evident in chromophores 4a-b that the incorporation of an azomethine (Schiff base) group, in which π-conjugation is enhanced by the incorporation of a pyridinyl group, leads to an increase in the planarity of the molecules. This increase in planarity of the D-π-A system in chromophores 4a-b facilitates an efficient ICT process from the electron-donating amino group towards the electron-accepting pyridine group. This leads to an increased redshift in the absorption and fluorescence spectra of chromophores 4a-b compared to chromophores 2a-b and 3a-b. The spectral variations in chromophores 2a-b, 3a-b, and 4a-b clearly reveal that the rise in π-conjugation, along with the introduction of an extra chromophore unit as in chromophores 4a-b, are the key factors that contribute to the spectral properties of the molecules (Fig. 2).

Increased fluorescence in polar liquids indicates excited-state stability, which is due to improved solvation effects. Meanwhile, the dielectric characteristics of the solvent may control changes in the conjugate electronic structure, leading to variations in absorbance maxima between solvents. The chemical and photophysical characteristics of some photoreactive components may change in response to light exposure. Conjugate 2b, including a Schiff base with a single nitro and an *N*,*N*-diphenyl-amino moiety, has an absorbance maximum at 396 nm in DMSO, most likely due to π–π* transitions (Fig. 3). This study carefully checked the absorbance and fluorescence of the newly synthesized benzimidazole hybrids 2a, 2b, 4a, and 4b in different organic solvents. As shown in Table S1, the interactions between the solvents greatly changed their photophysical properties.

### 3.3. *In vitro* cytotoxic assay

The cytotoxic effectiveness of the prepared benzimidazole hybrids 2a, 2b, 4a, and 4b was compared with that of sorafenib (standard drug) (Fig. 4 and Table S1). Hybrid 2a exhibited strong cytotoxicity against HepG2 cells (IC_50_ = 16.90 ± 0.36 µM), which suggests that it could be used to treat liver cancer. It exhibited low activity against PC3 cells (IC_50_ = 25.39 ± 0.02 µM), showing low effectiveness in treating prostate cancer. It showed moderate activity towards MCF-7 cells (IC_50_ = 18.14 ± 0.06 µM), showing some potential for curing breast cancer; IC_50_ = 54.27 ± 0.41 µM against WI-38 cells indicated low toxicity towards non-cancerous cells, which is beneficial. However, chromophore 2b exhibited an improvement in the cytotoxic effectiveness in HepG2 cells, with IC_50_ = 14.71 ± 0.11 µM, compared with that of chromophore 2a. On the other hand, 2b worked well towards PC3 cells (IC_50_ = 18.56 ± 0.19 µM), which means that it was more effective in curing prostate cancer. It was much more effective against MCF-7 cells, with an IC_50_ = 8.67 ± 0.53 µM, which is close to the value obtained for the reference (sorafenib). Additionally, hybrid 4a was very effective in killing HepG2 cells (IC_50_ = 12.64 ± 0.29 µM), making it stronger than hybrid 2a. It also showed the best activity against PC3 cells (IC_50_ = 12.19 ± 0.30 µM), which was similar to the activity of sorafenib. Finally, it showed average activity against MCF-7 cells (IC_50_ = 20.07 ± 0.21 µM). In addition, its IC_50_ of 39.48 ± 0.23 µM against WI-38 cells indicates the best selectivity profile among the tested hybrids. Hybrid 4b exhibited moderate cytotoxicity towards HepG2 cells, with an IC_50_ = 18.54 ± 0.08 µM. It showed relatively strong activity against PC3 cells with an IC_50_ = 23.62 ± 0.07 µM, although it was less effective than hybrid 4a. It also confirmed potent activity against MCF-7 cells with an IC_50_ of 9.64 ± 0.02 µM, close to that of sorafenib. Furthermore, its IC_50_ of 59.34 ± 0.37 µM in WI-38 cells indicates acceptable selectivity towards non-cancerous cells.

In order to assess the therapeutic window of the synthesized benzimidazole hybrids, the selectivity index of each compound was calculated using the following formula: SI=IC_50_(WI-38)/IC_50_(cancer cell line) (Table S1). The analysis of the data obtained revealed that hybrids 2b and 4b had the best selectivity index against MCF-7 cell lines. The selectivity index for these compounds was found to be 7.73 and 6.15, respectively. This revealed a good cytotoxicity profile for these compounds with less toxicity to non-cancerous cell lines. On the other hand, compound 4a had a moderate selectivity index for the HepG2 and PC3 cell lines. It was found that the selectivity index of compound 4a ranged from 3.12 to 3.24. The selectivity index of all the benzimidazole hybrids revealed that these compounds selectively target cancer cell lines without harming non-cancerous cells.

### 3.4. Structure–activity relationship

Insights into the substitution designs of the aniline moiety and diversity in the core structures of the benzimidazole chromophores showed distinguishable trends. These play a meaningful role in determining their cytotoxic effectiveness towards HepG2 (liver cancer), PC3 (prostate cancer), MCF-7 (human breast cancer), and WI-38 (lung fibroblasts) normal cell lines. Chromophores with an *N*,*N*-dimethyl-substitution skeleton, such as the chromophores 2a and 4a, show moderate to high cytotoxicity. This indicates that the incorporation of the pyridin-4-ylmethyleneamino group enhances their activity. However, they are more toxic against normal cells but maintain selectivity. Chromophores with an *N*,*N*-diphenyl substitution skeleton, such as chromophores 2b and 4b, exhibit higher cytotoxic effectiveness, indicating a loss of considerable activity, most likely due to the introduced bulk moiety, such as the pyridin-4-ylmethyleneamino group. However, the diphenyl chromophore 2b displays more cytotoxic effectiveness than the dimethyl chromophore 2a, possibly due to higher lipophilicity towards the cell targets. Moreover, the presence of the pyridin-4-ylmethyleneamino group in chromophores 4a and 4b increases their cytotoxic effectiveness, predominantly in HepG2 and PC3 cells, thereby increasing the potential of the molecules to engage in cancer cell-specific pathways while simultaneously increasing selectivity towards normal cells. The nitro group on the benzimidazole ring contributes to cytotoxicity, possibly *via* electron-withdrawing effects that stabilize interactions with nucleophilic sites in cellular proteins or DNA, explaining the higher activity observed in 2a and 2b compared to that observed in the unsubstituted analogues. Generally, the SAR analysis indicates that a combination of steric effects, electronic modulation by the amino substituents, and the incorporation of the pyridinyl Schiff base significantly influences selective cytotoxicity towards cancer cells while minimizing toxicity to normal cells.

### 3.5. *In vitro* VEGFR-2 kinase activity

**Fig. 5:**
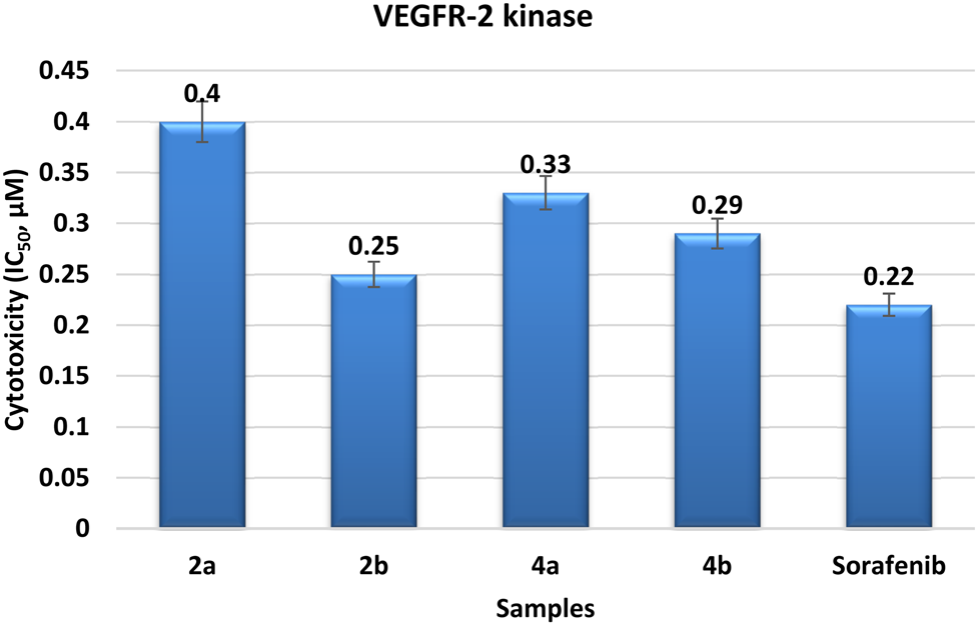
Cytotoxicity of the benzimidazole hybrids against VEGFR-2.

The study took into consideration the potency of our synthesized benzimidazoles 2a, 2b, 4a, and 4b as inhibitors of VEGFR-2 to explore possible anticancer therapy through anti-angiogenesis. However, chromophore 2b was found to be the most potent inhibitor (Table S2 and Fig. 5). It exhibited an inhibition activity (IC_50_ = 0.25 ± 0.18 µM) similar to that of the well-known VEGFR-2 inhibitor sorafenib (IC_50_ = 0.22 ± 0.09 µM). This result proves that chromophore 2b can be developed into a VEGFR-2 inhibitor in the future. Chromophore 4a demonstrated strong inhibition effectiveness (IC_50_ = 0.33 ± 0.04 µM). The ratio between its selectivity and activity suggests a potential for future development. Chromophore 4b exhibited high inhibitory activity, with an inhibition effectiveness of IC_50_ = 0.29 ± 0.22 µM. Although considerably less active than chromophore 2b, 4b remains a promising option for future investigations. Finally, chromophore 2a showed modest inhibition effectiveness over inhibition activity (IC_50_ = 0.40 ± 0.34 µM). Despite being less powerful than the other hybrids, it nevertheless showed considerable VEGFR-2 inhibition, indicating possible anti-angiogenic effects. Generally, the results show that the synthesized hybrids, especially 2b and 4a, show a lot of promise as VEGFR-2 inhibitors. This implies a need for further study in the context of anti-angiogenic cancer therapy.

### 3.6. Molecular docking

**Fig. 6:**
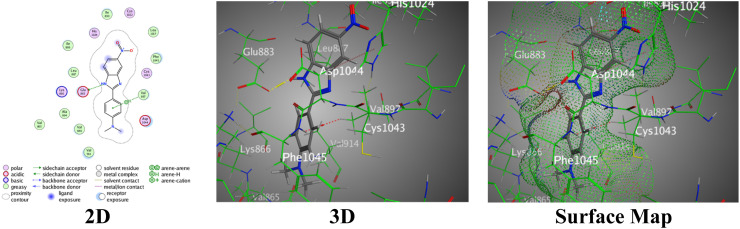
Images of the binding between hybrid 2a and PDB:2OH4.

Molecular docking was used to discover bindings between the synthesized benzimidazole ligands and the VEGFR-2 enzyme represented by the PDB:2OH4 protein (Table 1). The protein used for molecular docking is the PDB:2OH4 protein because it is the VEGFR-2 kinase enzyme, which is a proven target in the development of anticancer drugs that inhibit angiogenesis. The VEGFR-2 enzyme is a key mediator of angiogenesis in tumors. Nitrobenzimidazole with *N*,*N*-dimethylaniline 2a exhibited a weak binding score = −6.5681 and RMSD = 1.1964. It designed one H-bond between the N11 atom of the benzimidazole ring and Glu883, and a π–H bond between the *N*,*N*-dimethyl aniline ring and Val897 within intermolecular distances of 2.73 Å and 4.12 Å, respectively, reinforced by specific bindings that contribute to the complex stability (Fig. 6).

**Table 1:** Docking results of the synthesized benzimidazole 2a, 2b, 4a, and 4b hybrids

Hybrids	Binding affinity “S”	RMSD	Residual bindings	Binding types	Distance
2a	−6.5681	1.1964	N11 of benzimidazole ring with Glu883	H-donor	2.73
			*N*,*N*-dimethyl aniline ring with Val897	π-H	4.12
2b	−7.3457	1.3126	N11 of benzimidazole ring with Glu883	H-donor	3.07
			*N*,*N*-diphenyl aniline ring with Glu883	π-H	3.88
			Imidazole ring with Asp1044	π-H	3.91
4a	−7.2609	0.8048	N14 of benzimidazole ring with Cys1043	H-acceptor	3.38
			N14 of benzimidazole ring with Asp1044	H-acceptor π-H	3.01
			Imidazole ring with Val897		3.78
4b	−7.7633	1.3729	N14 of benzimidazole ring with Cys1043	H-acceptor	3.43
			N14 of benzimidazole ring with Asp1044	H-acceptor	3.45
			Pyridine ring with Leu838	π-H	4.04
			Imidazole ring with Val897	π-H	3.70
Sorafenib	−9.3024	1.9638	N12 of urea fragment with Glu883	H-donor	2.81
			N14 of urea fragment with Glu883	H-donor	2.89
			O15 of urea fragment with Asp1044	H-acceptor	3.03
			N26 of pyridine ring with Cys917	H-acceptor	3.71
			Pyridine ring with Leu838	π-H	4.50

**Fig. 7:**
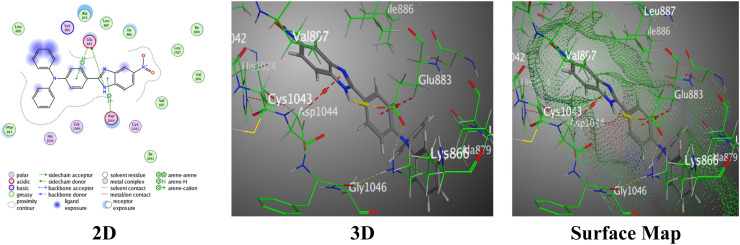
Images of the binding between hybrid 2b and PDB:2OH4.

In contrast, the nitrobenzimidazole analogue with an *N*,*N*-diphenylamine entity (2b) showed a stronger binding affinity with a docking score of −7.3457 and an RMSD value of 1.3126. This hybrid 2b involved multiple interactions as follows: H-bond between the N11 of the benzimidazole ring and Glu883, two π–H interactions between the *N*,*N*-diphenyl aniline ring and Glu883, and the imidazole ring with Asp1044 through interaction distances ranging from 3.07 Å to 3.91 Å (Fig. 7).

**Fig. 8:**
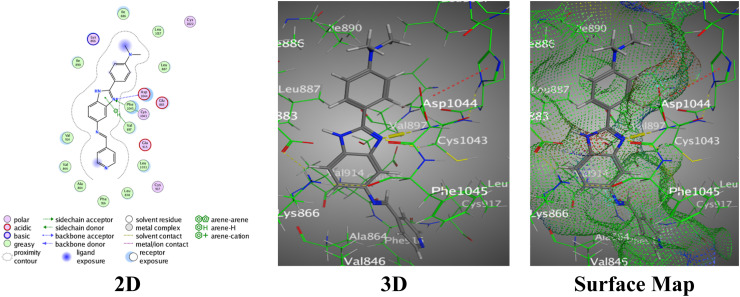
Images of the binding between hybrid 4a and PDB:2OH4.

The pyridinyl-benzimidazole with *N*,*N*-dimethylaniline 4a established a proper binding score = −7.2609 and lower RMSD = 0.8048, demonstrating a well-fitted binding interaction through two H-acceptors between the N14 of the benzimidazole ring with both Cys1043 and Asp1044. The imidazole ring is joined through a π–H interaction with Val897 over interaction distances of 3.01 Å to 3.78 Å, reflecting effective bindings with the essential PDB:2OH4 residues (Fig. 8).

**Fig. 9:**
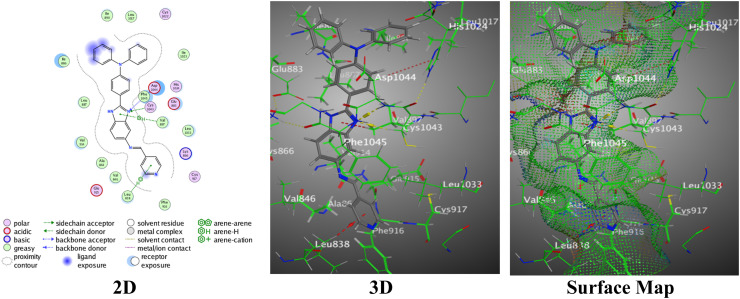
Images of the binding between hybrid 4b and PDB:2OH4.

Moreover, the pyridinyl-benzimidazole derivative containing the *N*,*N*-diphenylamine functional group (4b) proved to be the most potent based on its most favorable binding affinity among the hybrids, indicated by the docking score of −7.7633, accompanied by an RMSD of 1.3729. This hybrid 4b designed two H-acceptors amongst the N14 atom of the benzimidazole ring with Cys1043 and Asp1044, similar to hybrid 4a. In addition, π–H bonds were detected between the pyridine ring and Leu838, and the imidazole ring and Val897, completed by interaction distances of 3.43 Å to 4.04 Å (Fig. 9).

**Fig. 10:**
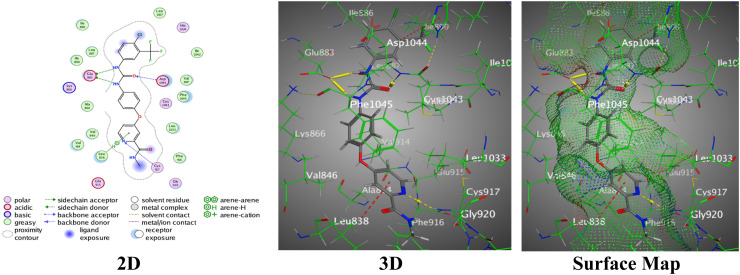
Images of the binding between sorafenib and PDB:2OH4.

Sorafenib (standard drug) had the uppermost binding score = −9.3024 and RMSD = 1.9638, demonstrating effective binding with the targeted PDB:2OH4 protein. It involved multiple bindings, counting H-bonds (two H-donors and two H-acceptors) with Glu883, Asp1044, and Cys917 in addition to one π–H interaction with Leu838, over interaction distances of 2.81 Å to 4.50 Å (Fig. 10).

However, the benzimidazole and *N*,*N*-dimethyl aniline rings found in all the newly synthesized benzimidazole hybrids 2a, 2b, 4a, and 4b create diverse H-bonds and π–H interactions, coupling with the PDB:2OH4 residues. In two- and three-dimensional images, several substituents of the greater pocket size of 2OH4 have a convincing binding score (Glu883, Val897, Asp1044, and Cys1043), delivering an appropriate cavity for the prepared benzimidazole hybrids.

### 3.7. Pharmacokinetic study

The SwissADME test on the man-made benzimidazole hybrids 2a, 2b, 4a, and 4b showed information about how they work in the body. However, hybrid 2a showed a lower M. wt. = 282.3 g mol^−1^ and iLog *P* = 1.86, indicating its sensible lipophilicity, which is beneficial for drug absorption (Table 2). Hybrid 2a set up a balanced H-bonding potential with three H-acceptors, three H-rotatable, and one H-donor bond. This made it a good candidate for interacting with biological membranes. The topological polar surface area (TPSA = 77.74 Å^2^) suggests that the membranes should be able to let the drug through properly. Hybrid 2a did not break Lipinski's rule of five, which means that it should be bioavailable when taken by mouth. This is supported by the fact that it did not absorb orally and had a bioavailability score of 0.55.

**Table 2:** SwissADME results of the synthesized benzimidazole 2a, 2b, 4a, and 4b hybrids^a^

Hybrids	M. wt	iLoglipophilicity (iLog *P*)P	HBA	HBD	TPSA	RT	Lipinski violations	Bioavailability score	GI absorption	BBB permeant	Pgp substrate
2a	282.3	1.86	3	1	77.74	3	0	0.55	High	Yes	No
2b	406.44	3.07	3	1	77.74	5	1	0.55	Low	No	No
4a	341.41	2.49	3	1	57.17	4	0	0.55	High	Yes	Yes
4b	465.55	3.84	3	1	57.17	6	1	0.55	Low	No	No

a M. wt.: molecular weight, iLog *P*: lipophilicity parameter, HBA: hydrogen bond acceptor, HBD: hydrogen bond donor, TPSA: topological polar surface area, and RT: rotatable bonds.

**Fig. 11:**
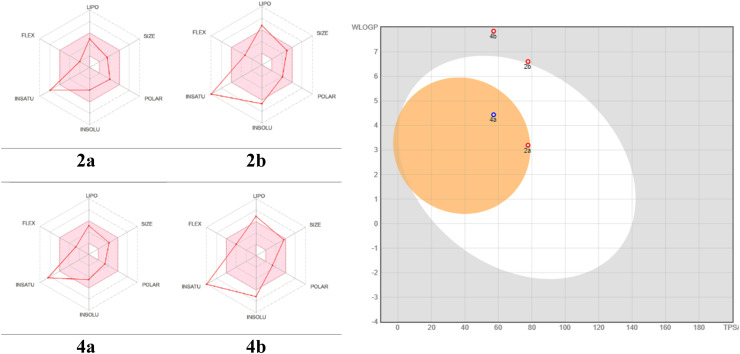
Radar . diagrams of the prepared hybrids with the corresponding boiled egg plot.

Hybrid 2a demonstrated potential activity in the central nervous system (CNS) without posing any risk to efflux-related bioavailability distributions. Meanwhile, hybrid 2b had a higher M. wt. of 406.44 g mol^−1^ with iLog *P* = 3.07, demonstrating a high lipophilicity similar to that of hybrid 2a. Despite having three H-acceptors, five H-rotatable, and one H-donor bond in common, hybrid 2b's bioavailability may be impacted. Still, hybrid 2b showed one of Lipinski's rules due to its higher molecular weight, which could undesirably impact its oral bioavailability. The low GI absorption and bioavailability score (0.55) confirm this. Furthermore, hybrid 2b was not permeant to the BBB, limiting its potential for CNS activity, which is a favorable attribute for reducing efflux and improving bioavailability. It also had a good M. wt. = 341.41 g mol^−1^ and iLog *P* = 2.49, showing moderate lipophilicity through the same H-donor, H-acceptors and three rotatable bonds with lower TPSA = 57.17 Å^2^. This helped the membrane permeability improve compared to the other hybrids. Similar to hybrid 2a, hybrid 4a did not break any of Lipinski's rules, demonstrating good oral bioavailability, which was supported by its high GI absorption. Outstandingly, hybrid 4a was permeant to the BBB, representing potential for CNS activity. In addition, hybrid 4b had the highest molecular weight (M. wt. = 465.55 g mol^−1^) compared to the others, and its iLog *P* value was 3.84, which means that it was very lipophilic. This could have an effect on its absorption profile through three H-acceptors, six H-rotatable, and one H-donor bond, which means it was more flexible. This may have led to its low GI absorption over TPSA = 57.17 Å^2^, which is a good sign for membrane permeability. Despite its high molecular weight, hybrid 4b demonstrated one of Lipinski's rules, a factor that contributes to its low GI absorption. Hybrid 4b did not permeate the BBB, limiting its potential for CNS activity (Fig. 11).

## 4. Conclusion

The benzimidazole-based chromophores 2a and 2b are synthesized by the combination of 4-nitro-*o*-phenylenediamine and 4-formyl-*N*,*N*-dimethylanilin 1a and 4-formyl-*N*,*N*-diphenylanilin 1b, respectively. Whereas, the benzimidazole-pyridines 4a and 4b were developed by the reduction of both 5-nitrobenzimidazoles 2a and 2b tracked by the reaction of amidisonicotinic aldehyde with 5-amino-2-benzimidazoles 3a and 3b, respectively. It was revealed that the solvent environment had a significant impact on the photophysical behavior of the benzimidazole hybrids. In DMSO, conjugate 2a exhibited absorbance and fluorescence maxima at 390 and 528 nm, respectively, while conjugate 2b had the maxima at 396 and 536 nm, respectively. Hybrids 4a and 4b exhibited similar solvent-dependent activity. These results demonstrated that the enhancement of fluorescence in polar solvents for example, DMSO, indicates that solvent polarity stabilizes the excited state. The alterations in the maximum absorbance among solvents reflected electronic structure changes caused by solute–solvent interactions. These results highlight the significance of solvent effects in determining the photophysical characteristics of these molecules. However, the benzimidazole hybrids exhibited different levels of cytotoxicity against different types of cancer cells. Hybrid 4a was the most effective, especially against HepG2 (IC_50_ = 12.64 ± 0.29 µM) and PC3 (IC_50_ = 12.19 ± 0.30 µM) cells. Hybrid 2b also displayed strong cytotoxicity, especially against MCF-7 cells (IC_50_ = 8.67 ± 0.53 µM). All the hybrids demonstrated desirable selectivity, exhibiting low toxicity towards non-cancerous WI-38 cells. These findings suggest that hybrid 4a, in particular, holds promise as a potential anticancer agent. The synthesized benzimidazole hybrids demonstrated potent VEGFR-2 inhibitory activity, with inhibition activity values ranging from IC_50_ = 0.25 ± 0.18 µM to IC_50_ = 0.40 ± 0.34 µM. Hybrid 2b was identified as the most powerful inhibitor, comparable to sorafenib (reference). The results suggest that these hybrids, chiefly hybrid 2b, hold significant potential as VEGFR-2 inhibitors, warranting further investigation in the development of anti-angiogenic medicines. Hybrids 2a and 4b had moderate inhibitory activity, with inhibition activities of IC_50_ = 0.40 ± 0.34 µM and IC_50_ = 0.29 ± 0.22 µM, respectively. The results show that the hybrids, especially 2b and 4a, could be good candidates for further development as VEGFR-2 inhibitors. This could lead to new ways of treating cancer by stopping blood vessels from growing. Moreover, a molecular docking investigation found that the synthesized benzimidazole hybrids had significant interactions with the target PDB:2OH4 protein, with hybrid 4b yielding the most promising findings. Its docking score of −7.7633, along with strong contacts with critical residues, implies that 4b might be a promising candidate for future development as an inhibitor. The way that these hybrids bind and interact with other molecules, especially with Cys1043, Asp1044, and Val897, corresponds to how well they block activity. Additionally, the pharmacokinetic properties of the synthesized hybrids were studied *via* SwissADME. Hybrids 2a and 4a displayed good ADME profiles, with high GI absorption and BBB permeability. This means that they are likely to be good candidates for further development as drugs that can be taken by mouth and may have CNS activity. Although the present study has been focused on benzimidazole hybrids based on the use of isonicotinaldehyde, the scope of future studies will be to extend the library of compounds by employing a wide variety of aldehydes, including α,β-unsaturated aldehydes, for example, cinnamaldehydes, along with other heterocyclic aldehydes like indole-3-aldehyde, thiophene-2-aldehyde, and pyrrole-2-aldehyde. Such studies are expected to provide better insights into structure–property relationships, which would ultimately help to understand the effects of the substituents on the photophysical, anticancer, and VEGFR-2 inhibitory properties of the benzimidazole-based chromophores.

## Consent to participate

All authors participated in this work.

## Consent to publish

All authors agreed to publish.

## Conflicts of interest

The authors declare that they have no competing interests.

## Supplementary Material

RA-016-D6RA00254D-s001

## Data Availability

The data that support the findings of this study are available from the corresponding author upon reasonable request. Supplementary information (SI) is available. See DOI: https://doi.org/10.1039/d6ra00254d.
