# Supplementary material for: Novel donor-π-acceptor benzimidazole-based chromophores: synthesis, antitumor assessment, and pharmacokinetics
Source: RSC Adv. 2026 Mar 11;16(15):13644–57. doi: 10.1039/d6ra00254d (PMC12977370; doi:10.1039/d6ra00254d)
Supplement: RA-016-D6RA00254D-s001 [file RA-016-D6RA00254D-s001.pdf]

Supplementary Information (SI) for RSC Advances.
This journal is © The Royal Society of Chemistry 2026

Supporting Information

Novel Donor-π-Acceptor Benzimidazole-Based Chromophores: Synthesis, Antitumor Assessment, and Pharmacokinetics

Biological Evaluation

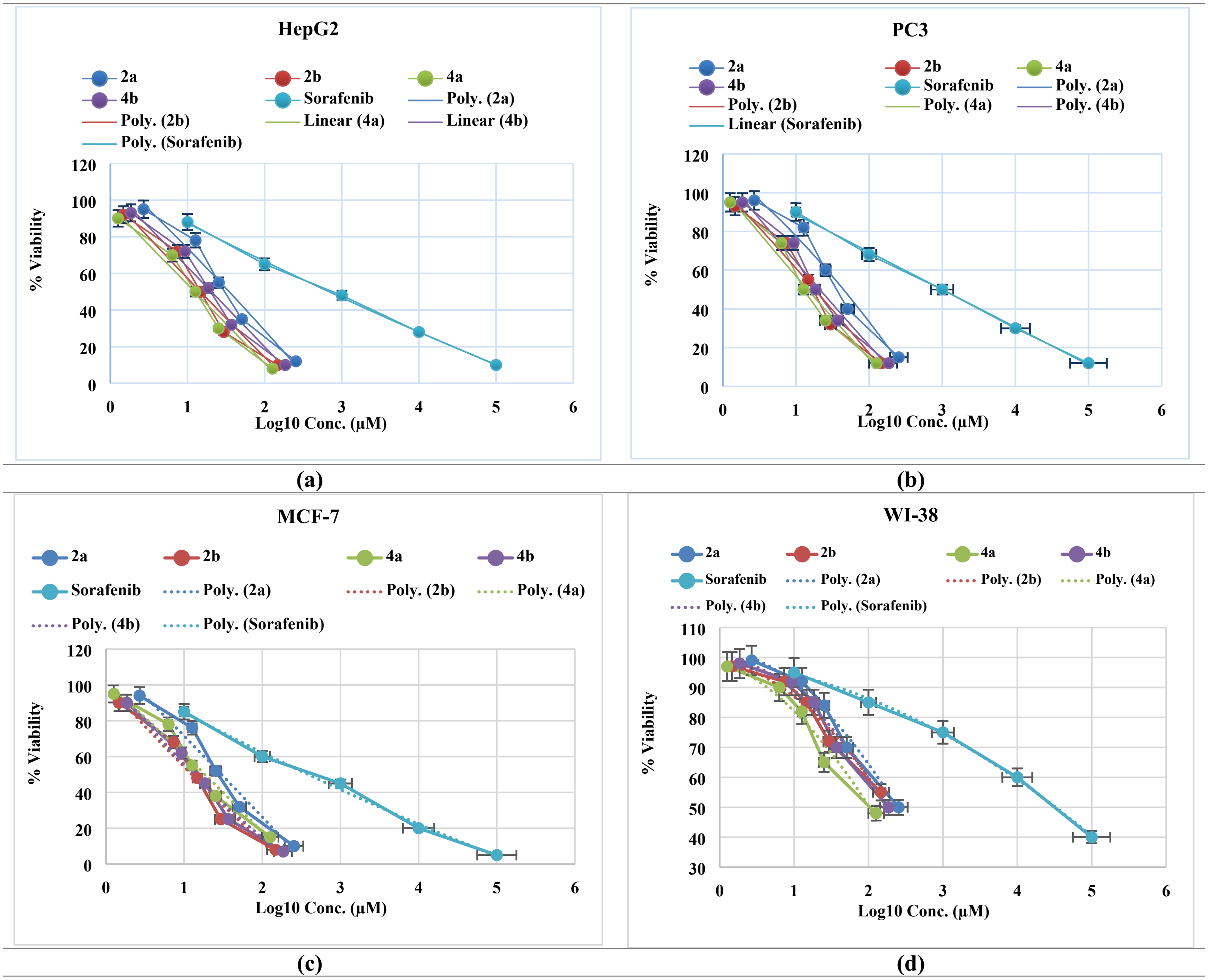

Figure S1. Dose-response curves showing the effect of compounds **2a**, **2b**, **4a**, **4b**, and Sorafenib on cell viability. Cell lines: (a) HepG2, (b) PC3, (c) MCF-7, and (d) WI-38. Data represent mean ± SD (n = 3) and are plotted against $\log_{10}$ inhibitor concentration (μM). Trendlines were added to visualize the dose-response relationship.

Cytotoxicity Assessment

The cells were cultured in RPMI-1640 medium with 10% fetal bovine serum. Antibiotics were added 100 units/mL penicillin and 100 μg/mL streptomycin at 37 °C in a 5% CO_2 incubator. The cells were seeded in a 96-well plate at a density of $1.0x10^4$ cells/well at 37 °C for 48 h under 5% CO_2. After incubation, the cells were treated with different concentration of compounds and incubated for 24 h. Discard the medium. Fixed with 10% trichloroacetic acid (TCA) 150 μL/well for 1h at 4 ºC. Wash by water 3 times (TCA) reduce SRB protein binding). Wells will be stained by SRB 70 μL/well for 10 min at room temperature with 0.4%. 70 μL/well and incubated in the dark. Wash with acetic acid 1% to remove unbound dye (end point: colorless drainage). The plates will be air dried 24 h. The dye will be solubilized with 50 μL/well of 10 m Mtris base (pH 7.4) for 5 min on a shaker at 1600 rpm.

Table S1. *In vitro* cytotoxic activity and selectivity of the synthesized benzimidazole hybrids

IC_{50} values (μM) represent the mean ± SD of three independent experiments. Selectivity index (SI) was calculated as SI = IC_{50} (WI-38)/IC_{50} (cancer cell line). Hill slope values were obtained from four-parameter logistic (4PL) fitting of the dose-response curves. Sorafenib was used as the reference drug.

Hybrid	IC_{50} (μM) HepG2	SI (HepG2)	IC_{50} (μM) PC3	SI (PC3)	IC_{50} (μM) MCF-7	SI (MCF-7)	IC_{50} (μM) WI-38	Hill slope
**2a**	16.90 ± 0.36	3.21	25.39 ± 0.02	2.14	18.14 ± 0.06	2.99	54.27 ± 0.41	**1.1**
**2b**	14.71 ± 0.11	4.56	18.56 ± 0.19	3.61	8.67 ± 0.53	7.73	67.03 ± 0.12	**1.0**
**4a**	12.64 ± 0.29	3.12	12.19 ± 0.30	3.24	20.07 ± 0.21	1.97	39.48 ± 0.23	**1.2**
**4b**	18.54 ± 0.08	3.20	23.62 ± 0.07	2.51	9.64 ± 0.02	6.15	59.34 ± 0.37	**1.1**
**Sorafenib**	9.38 ± 0.16	4.76	12.35 ± 0.08	3.62	8.13 ± 0.33	5.49	44.65 ± 0.05	**1.1**

In vitro VEGFR assay

In vitro VEGFR-2 tyrosine kinase activity was assayed using an enzyme-linked immunosorbent assay kit (Boehringer Mannheim, SA). In brief, ellagic acid was incubated with VEGFR-2 (Upstate) in assay buffer containing Mg^{2+} and ATP in a 96-well plate coated with a poly-Glu-Tyr substrate. Phosphorylated tyrosine was then detected by sequential incubation with a mouse IgG anti-phosphotyrosine antibody and an HRP-linked sheep anti-mouse immunoglobulin antibody. Color was developed with an HRP chromogenic

substrate and quantified by an ELISA reader at wavelength 450 nm. The results were expressed as percent kinase activity.

Table S2. *In vitro* VEGFR-2 inhibitory activity of the synthesized benzimidazole hybrids.
IC_{50} values represent the mean ± SD of three independent experiments (n = 3). Hill slope values were derived from 4PL nonlinear regression analysis of the dose-response curves.

Hybrid	VEGFR-2 IC_{50} (µM)	Hill slope
**2a**	0.40 ± 0.34	**1.0**
**2b**	0.25 ± 0.18	**1.1**
**4a**	0.33 ± 0.04	**1.0**
**4b**	0.29 ± 0.22	**1.0**
**Sorafenib**	0.22 ± 0.09	**1.0**

N.B. Statistical significance was determined using one-way ANOVA followed by Dunnett's post-hoc test versus Sorafenib ($p < 0.05$).

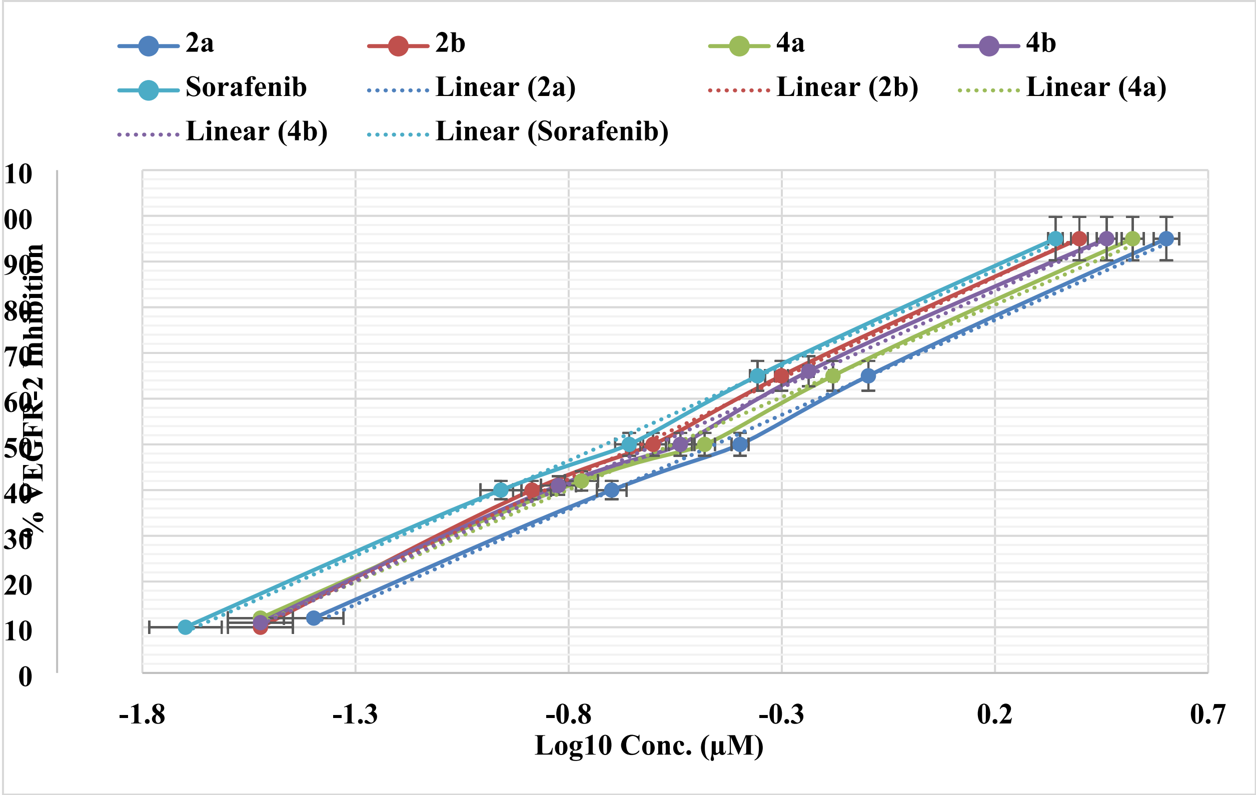

Figure S2. Dose-response curves for VEGFR-2 inhibition. Data represent mean ± SD (n = 3) plotted against $\log_{10}$ inhibitor concentration (µM).

Photophysical Properties

Table S3. The absorbance and fluorescence (λ_{max} nm) for the synthesized benzimidazole conjugates **2a, 2b, 4a,** and **4b** in diverse solvents.

**No.**		**Toluene**	**Dichloromethane (DCM)**	**Acetonitrile (ACN)**	**Ethanol**	**Dimethyl Sulfoxide (DMSO)**
**2a**	***Abs ($\varepsilon\times10^3$)***	336 (2.83)	348 (3.01)	372 (3.15)	388 (3.19)	390 (3.40)
	FL (ϕ)	497 (0.69)	507 (0.784)	514 (0.7461)	524 (0.8264)	528 (0.8652)
	$\Delta\bar{\nu}$ (cm^{-1})	9493	8872	7403	6671	6713
**2b**	***Abs ($\varepsilon\times10^3$)***	375 (3.47)	384 (3.66)	388 (3.70)	390 (3.88)	396 (3.92)
	FL (ϕ)	512 (0.71)	520 (0.7368)	527 (0.7592)	531 (0.760)	536 (0.7849)
	$\Delta\bar{\nu}$ (cm^{-1})	7279	6880	6735	6877	6735
**4a**	***Abs ($\varepsilon\times10^3$)***	389 (4.2)	395 (4.71)	401 (4.78)	398 (4.89)	408 (4.97)
	FL (ϕ)	520 (0.76)	532 (0.7884)	526 (0.7920)	536 (0.826)	539 (0.8491)
	$\Delta\bar{\nu}$ (cm^{-1})	6546	6578	5922	6499	6019
**4b**	***Abs ($\varepsilon\times10^3$)***	418 (4.32)	424 (4.43)	412 (4.71)	412 (4.59)	406 (4.94)
	FL (ϕ)	520 (0.77)	549 (0.7913)	546 (0.8038)	569 (0.820)	573 (0.8280)
	$\Delta\bar{\nu}$ (cm^{-1})	4677	5431	6011	6796	7245

Where; ε: $Lmol^{-1}cm^{-1}$; ϕ: Quantum yield; $\Delta\bar{\nu}$ stock shift

Spectral Charts

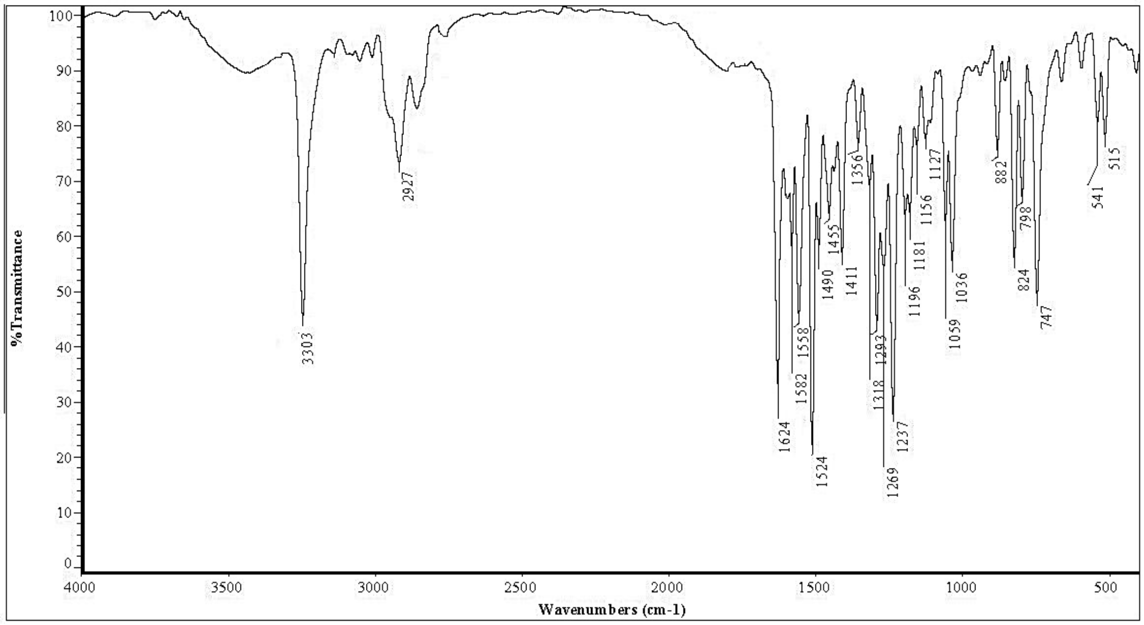

Figure S3. I.R spectrum of hybrid **2a**.

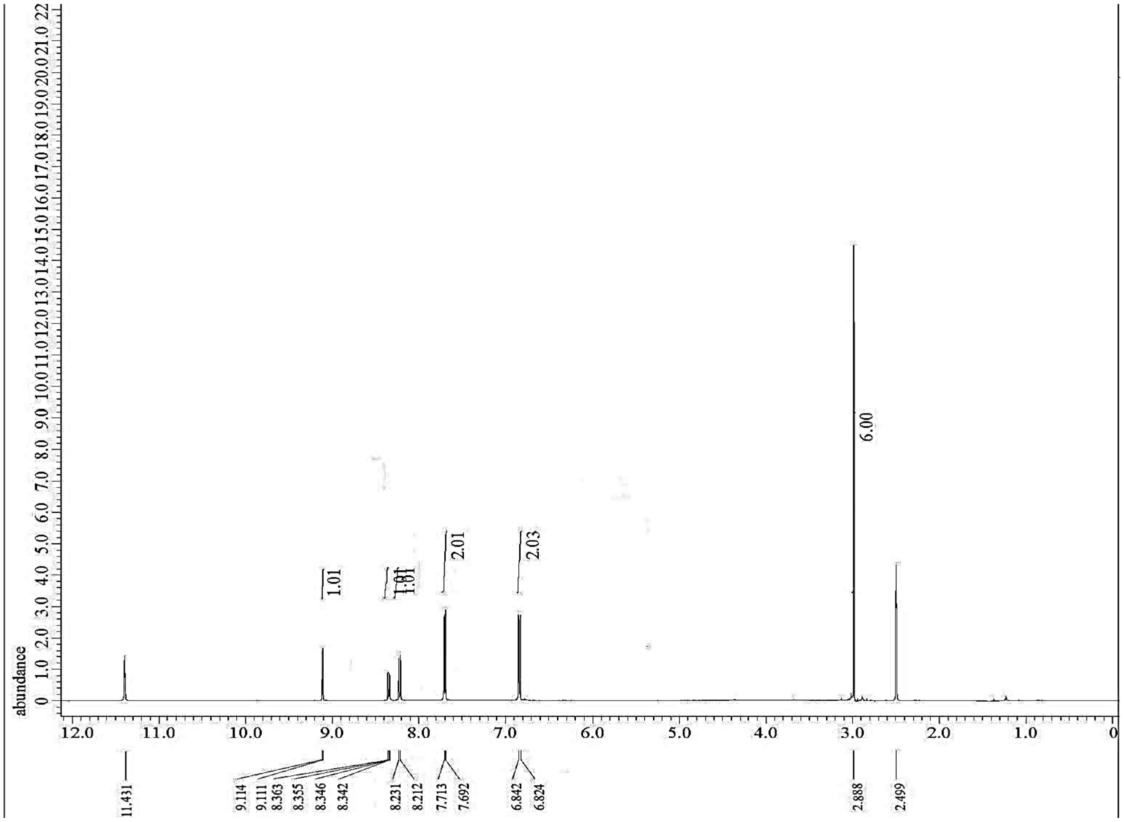

Figure S4. ^{1}H-NMR spectrum of hybrid **2a**.

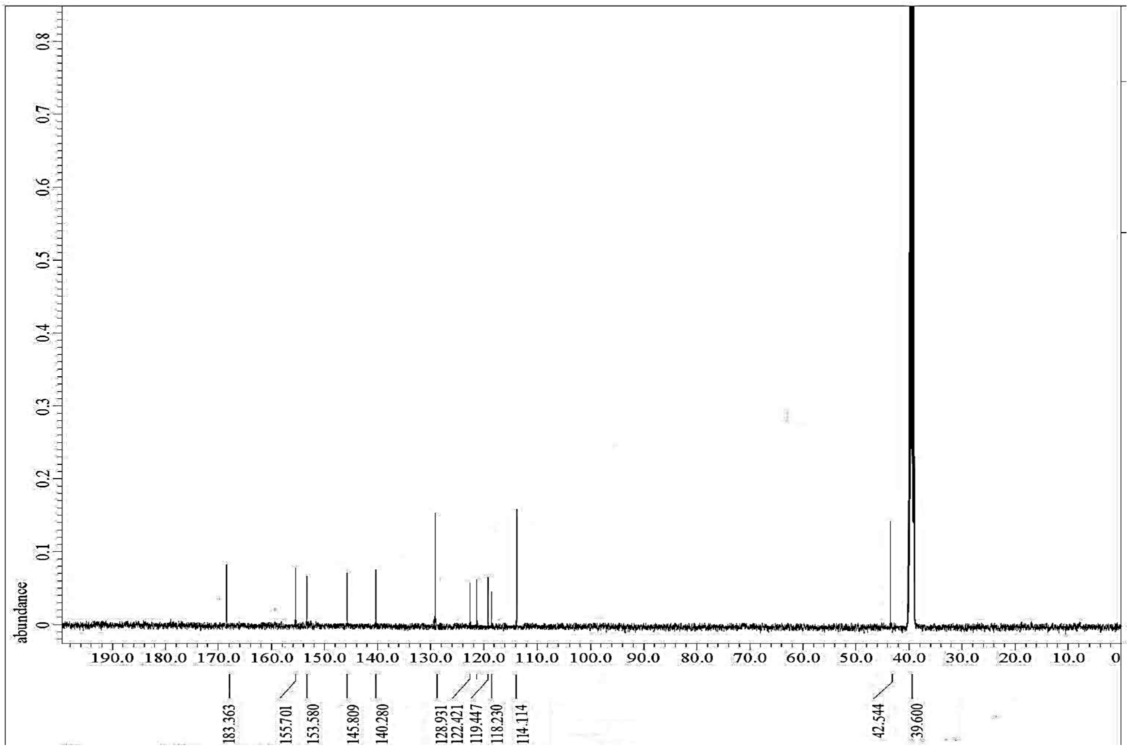

Figure S5. ^{13}C-NMR spectrum of hybrid **2a**.

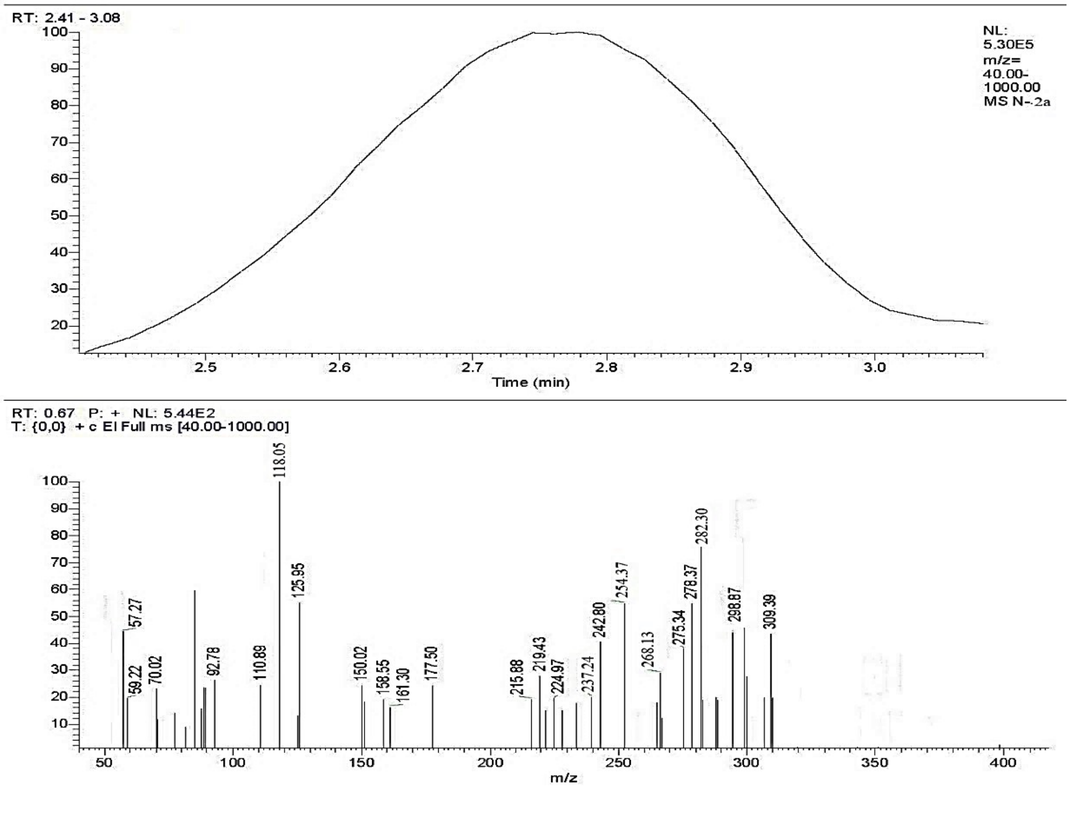

Figure S6. Mass analysis of hybrid **2a**.

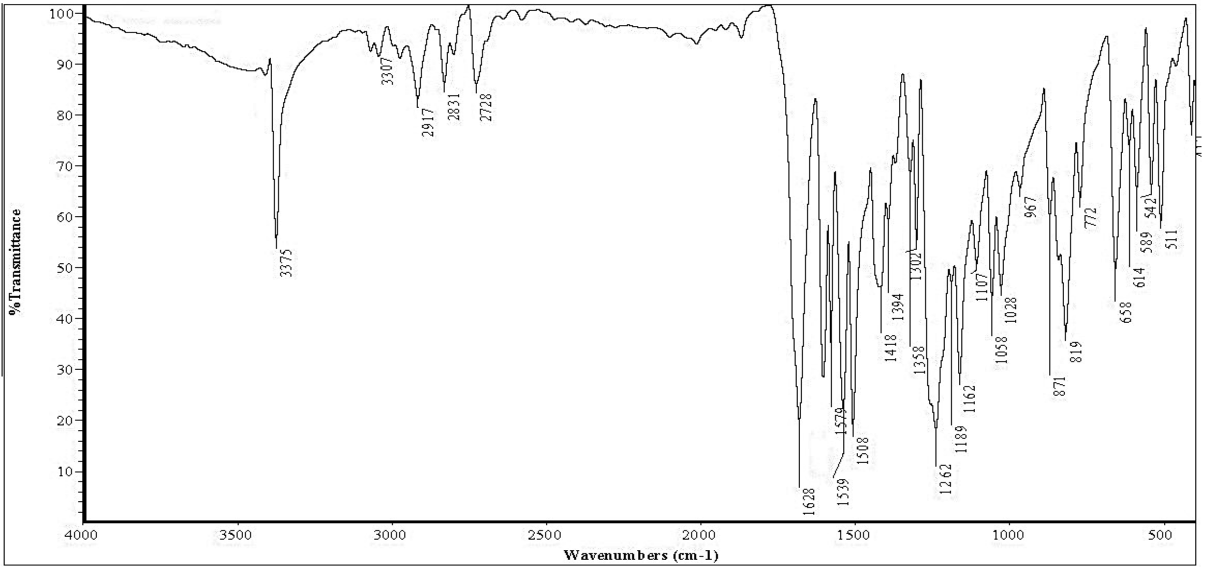

Figure S7. I.R spectrum of hybrid **2b**.

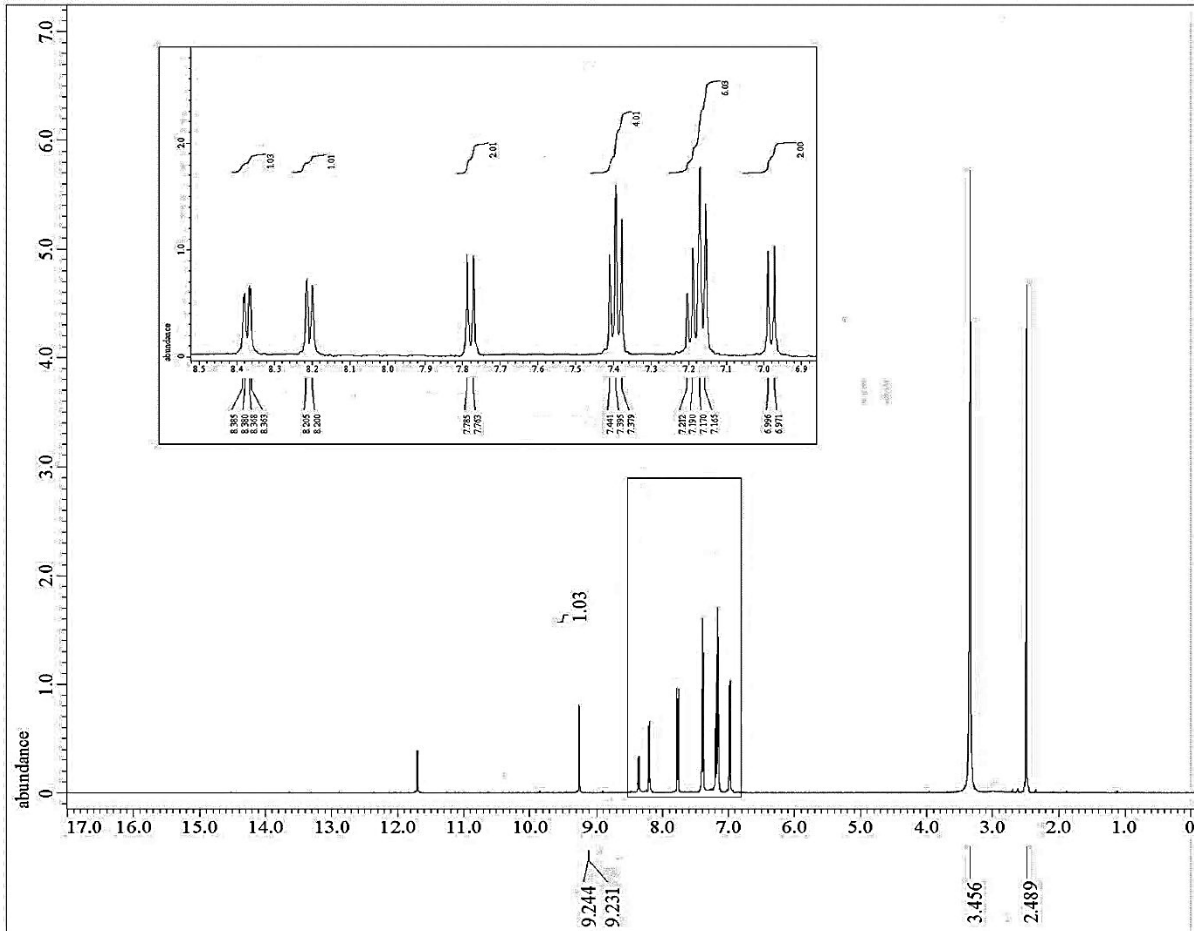

Figure S8. ^{1}H-NMR spectrum of hybrid **2b**.

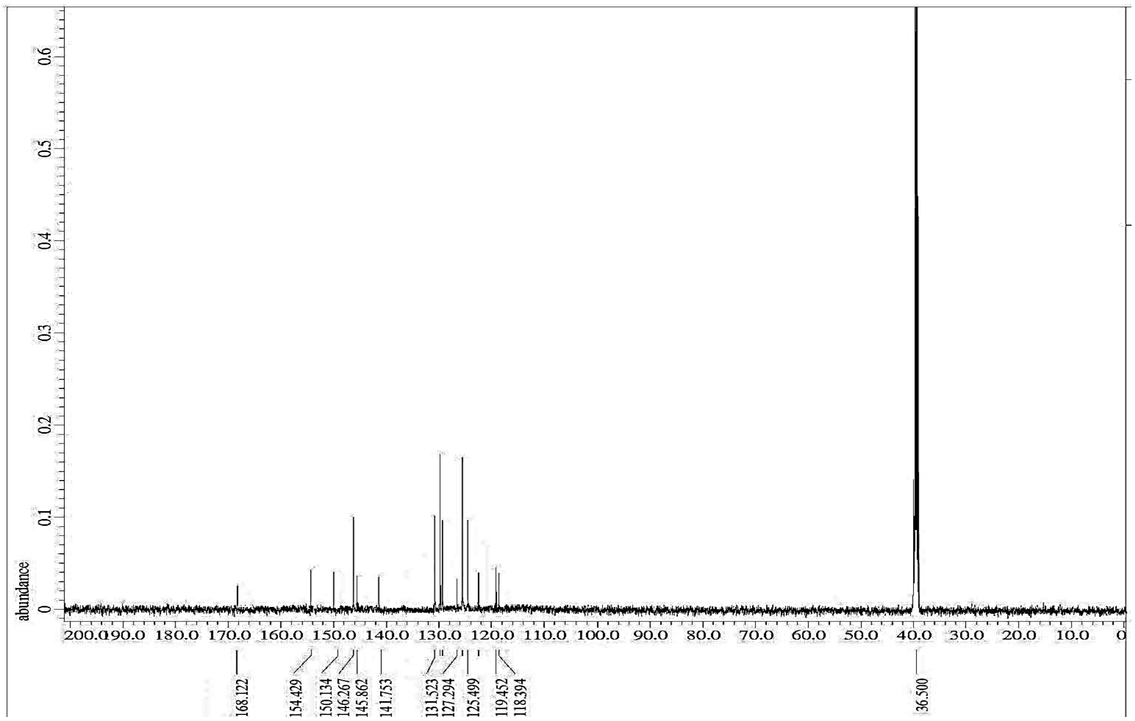

Figure S9. ^{13}C-NMR spectrum of hybrid **2b**.

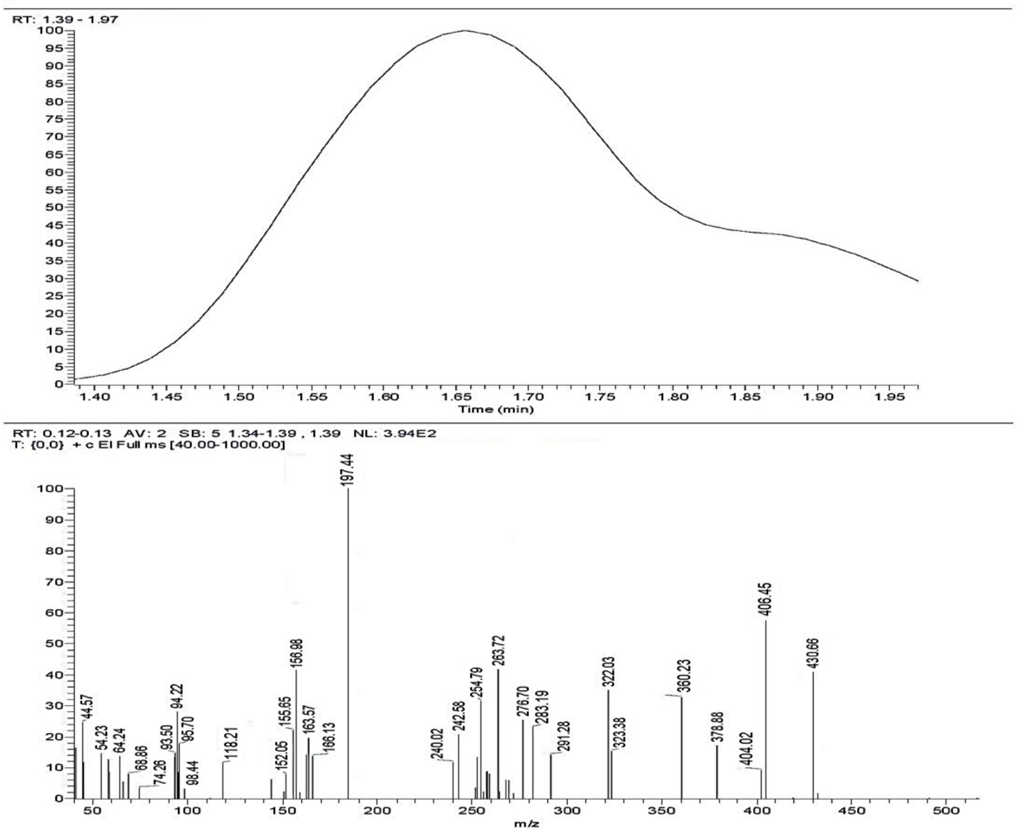

Figure S10. Mass analysis of hybrid **2b**.

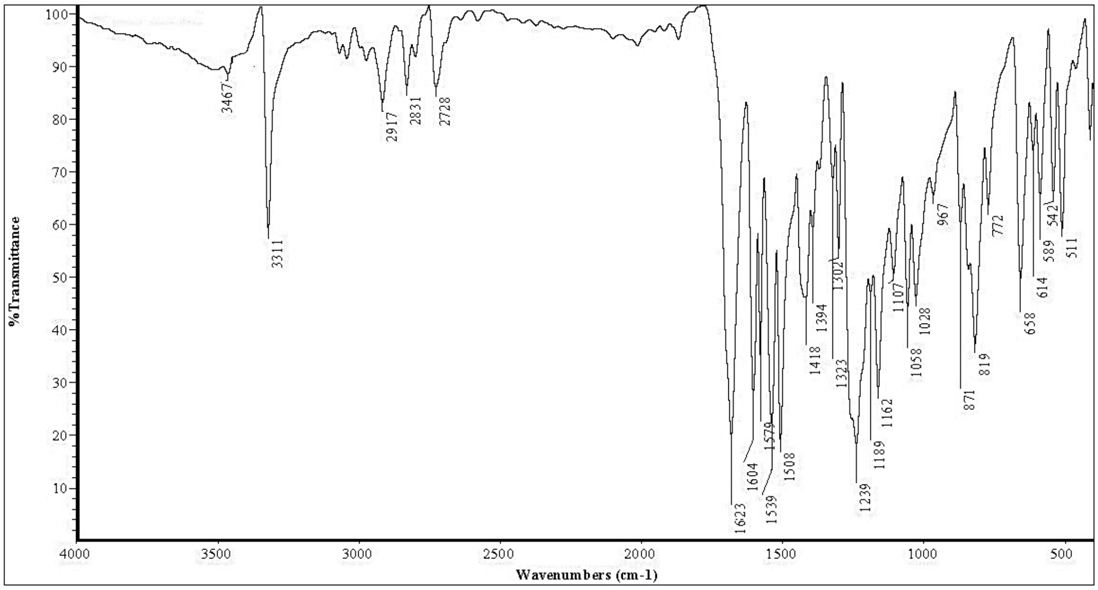

Figure S11. I.R spectrum of hybrid **3a**.

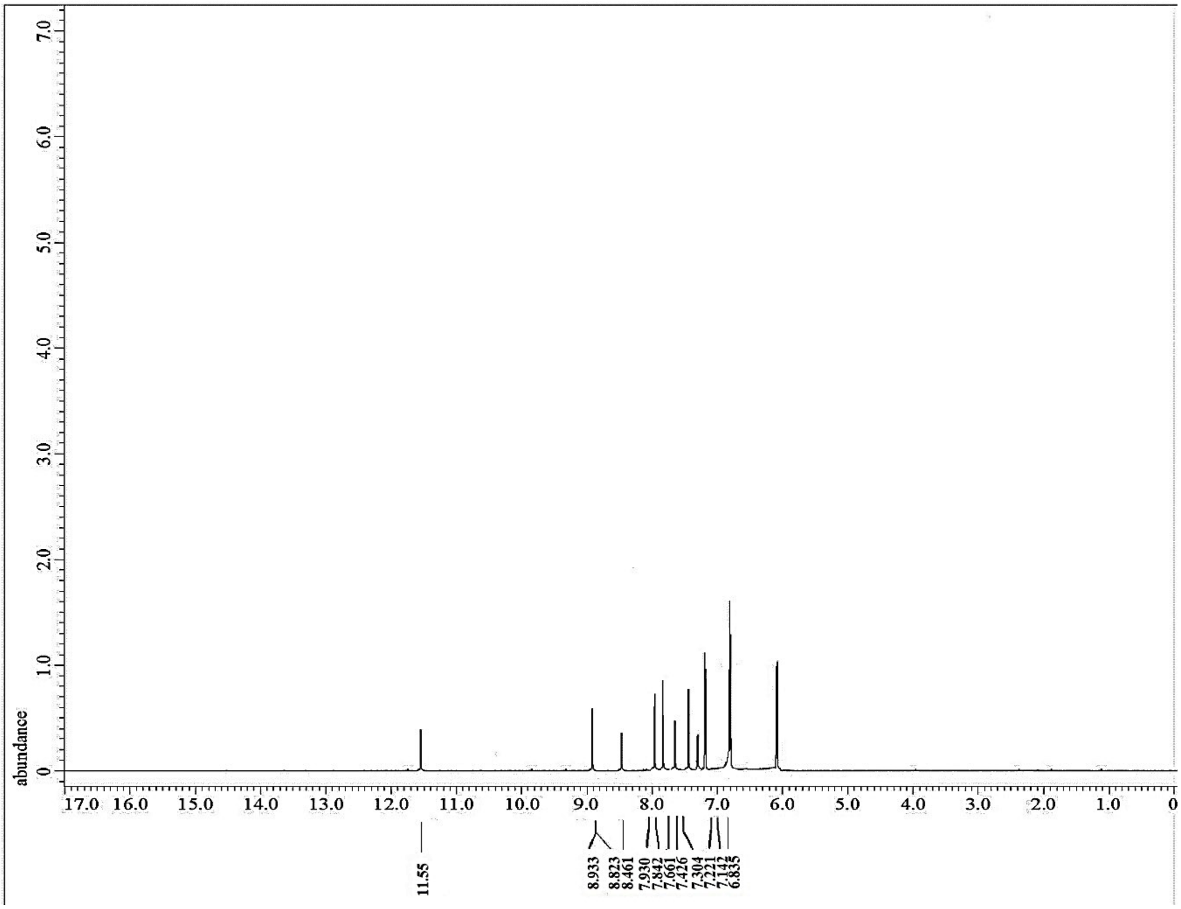

Figure S12. ^{1}H-NMR spectrum of hybrid **3a**.

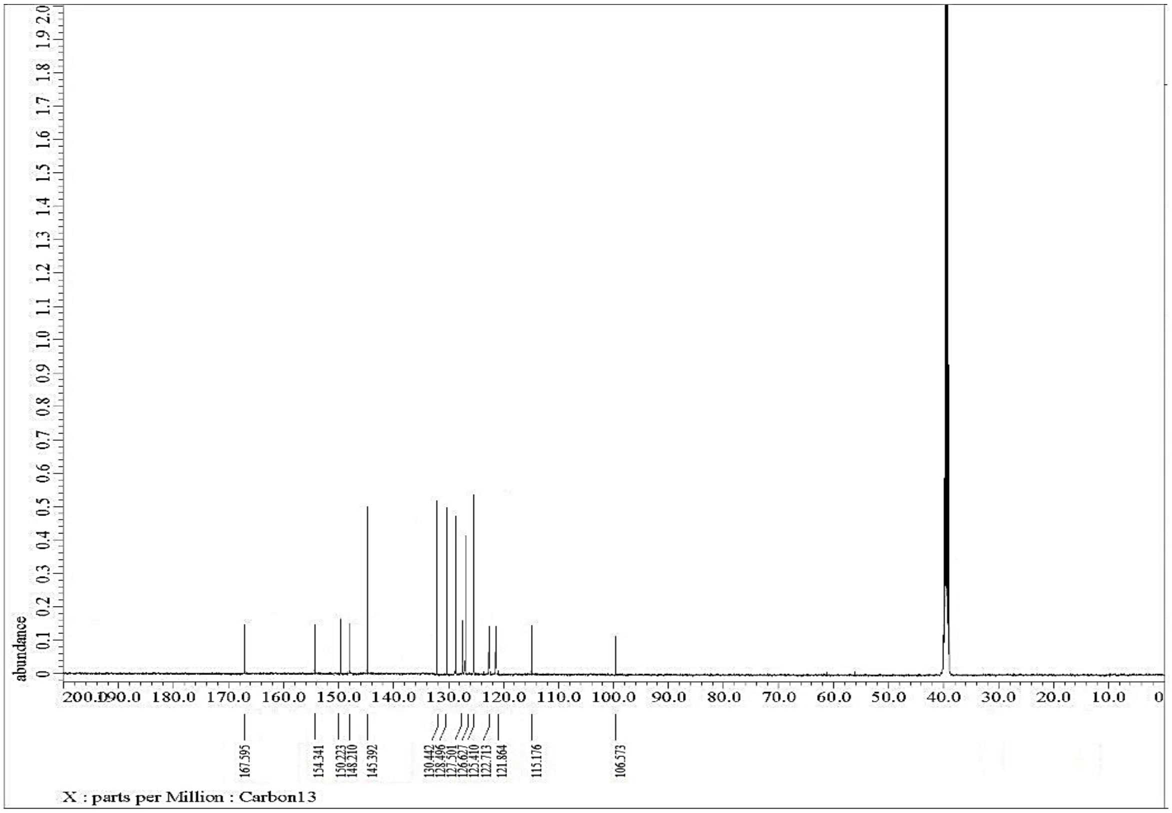

Figure S13. ^{13}C-NMR spectrum of hybrid **3a**.

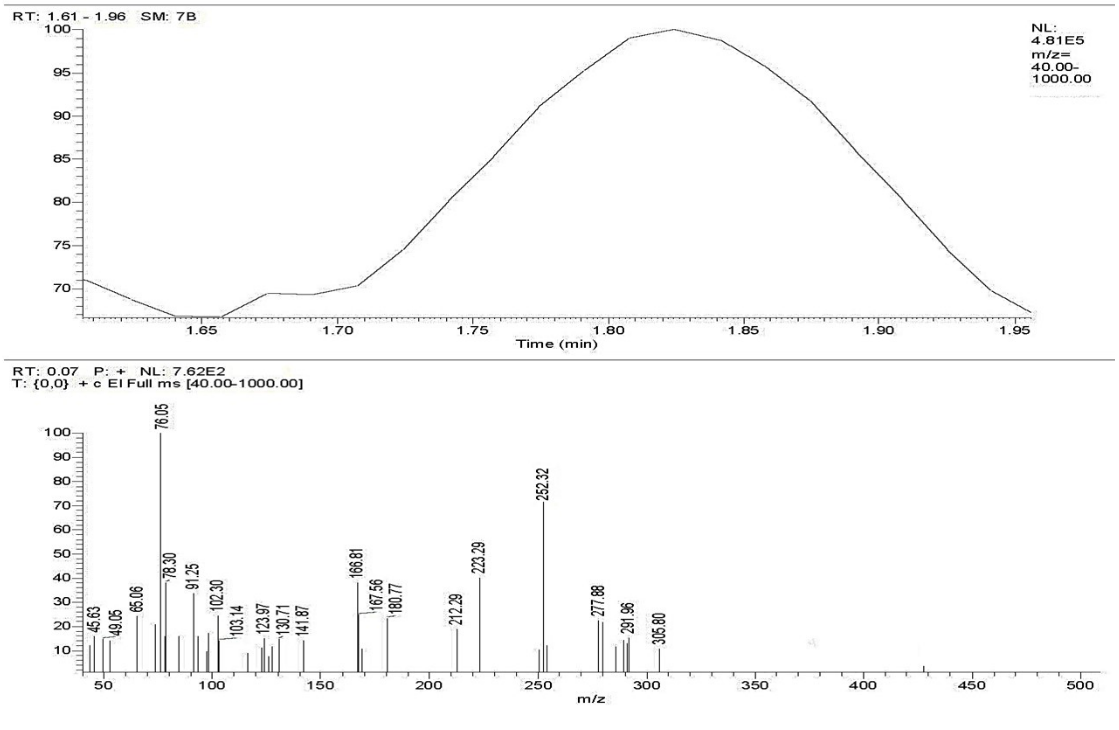

Figure S14. Mass analysis of hybrid **3a**.

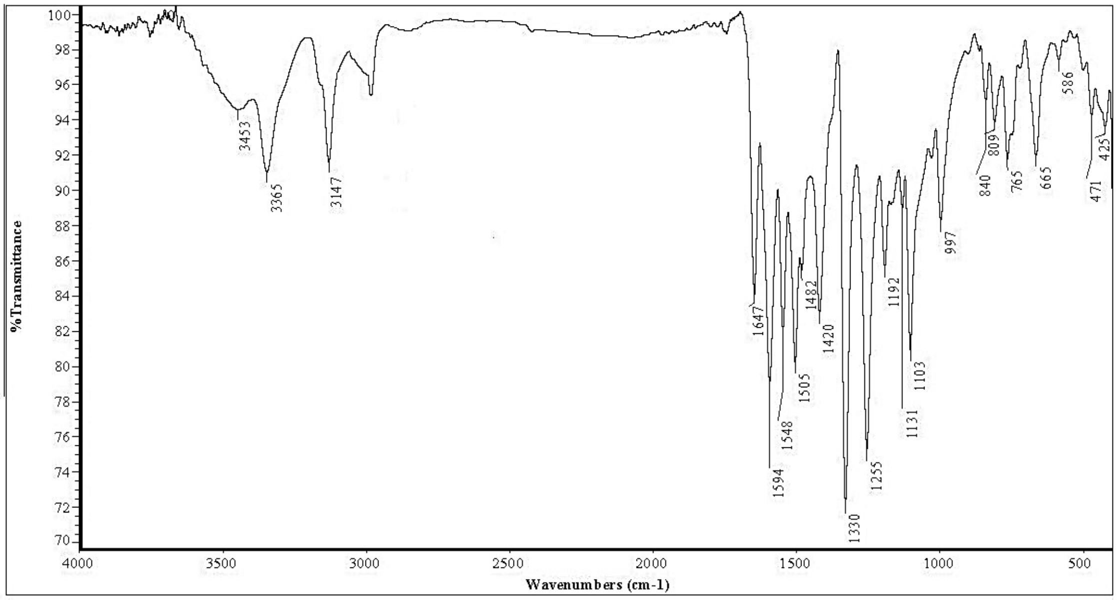

Figure S15. I.R spectrum of hybrid **3b**.

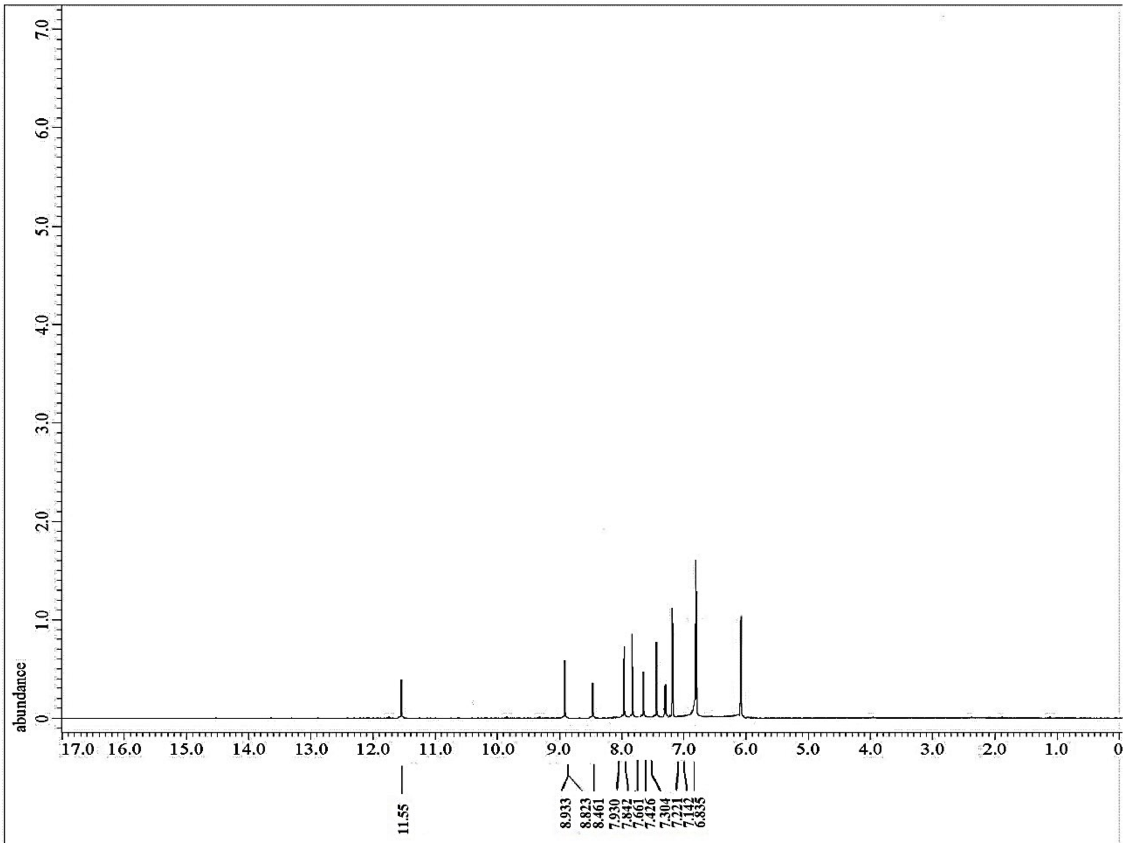

Figure S16. ^{1}H-NMR spectrum of hybrid **3b**.

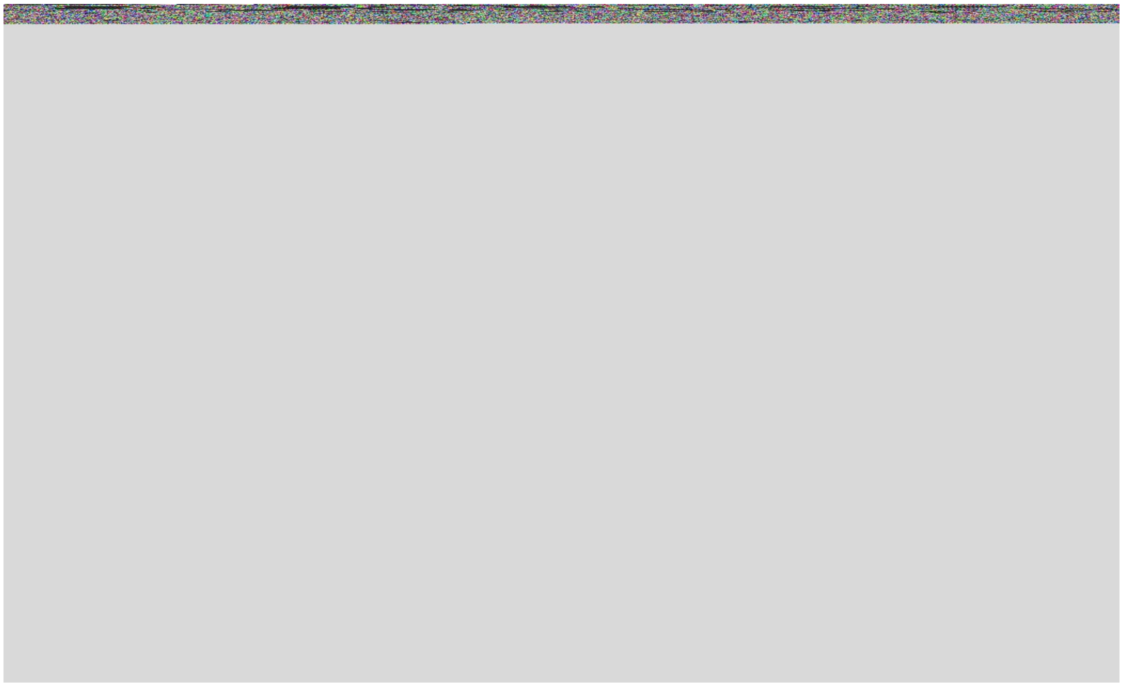

Figure S17. ^{13}C-NMR spectrum of hybrid **3b**.

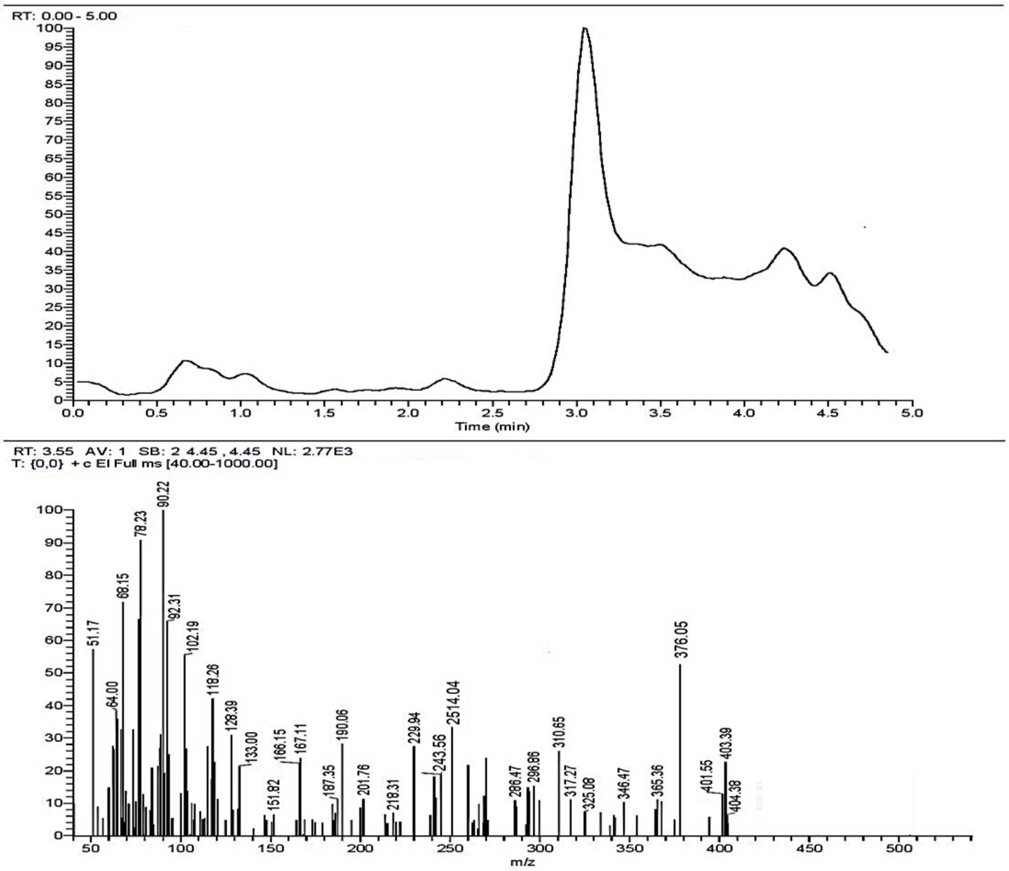

Figure S18. Mass analysis of hybrid **3b**.

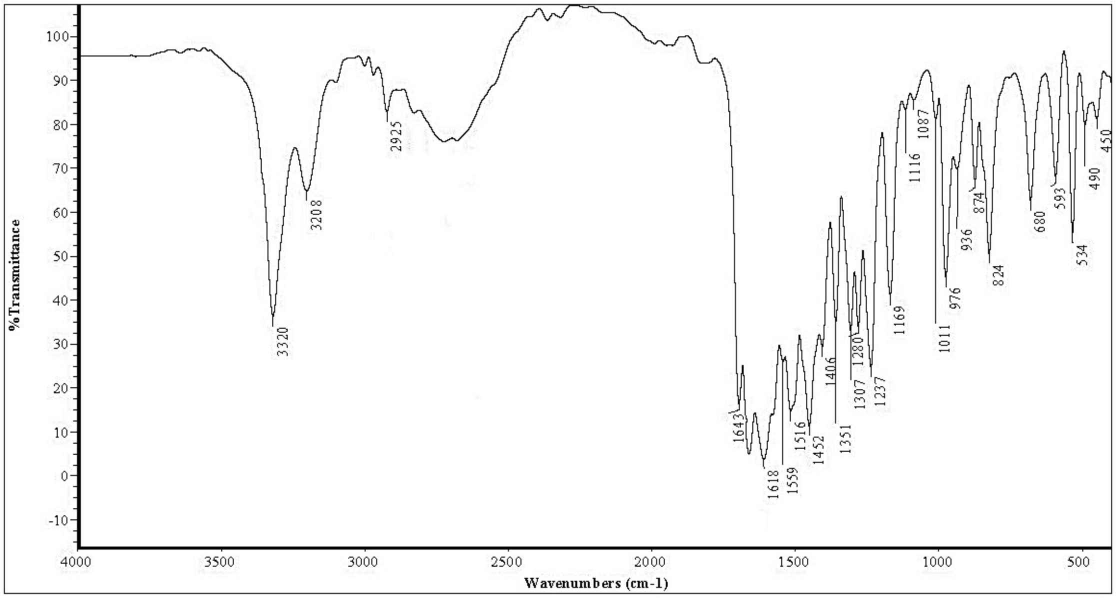

Figure S19. I.R spectrum of hybrid **4a**.

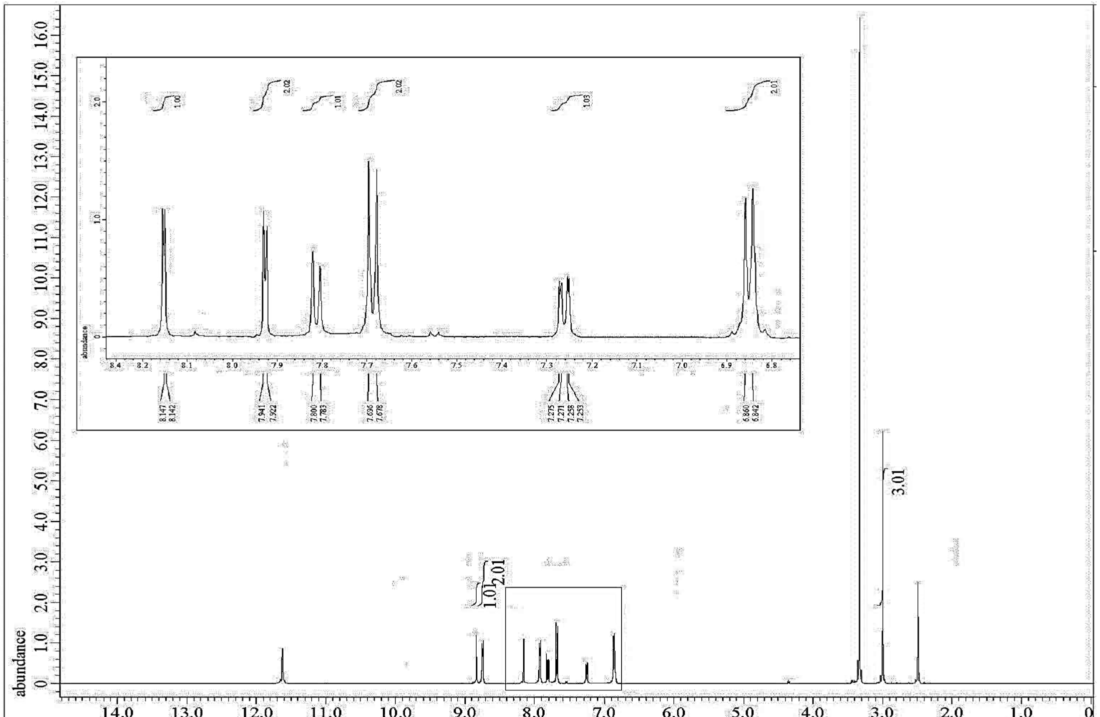

Figure S20. ^{1}H-NMR spectrum of hybrid **4a**.

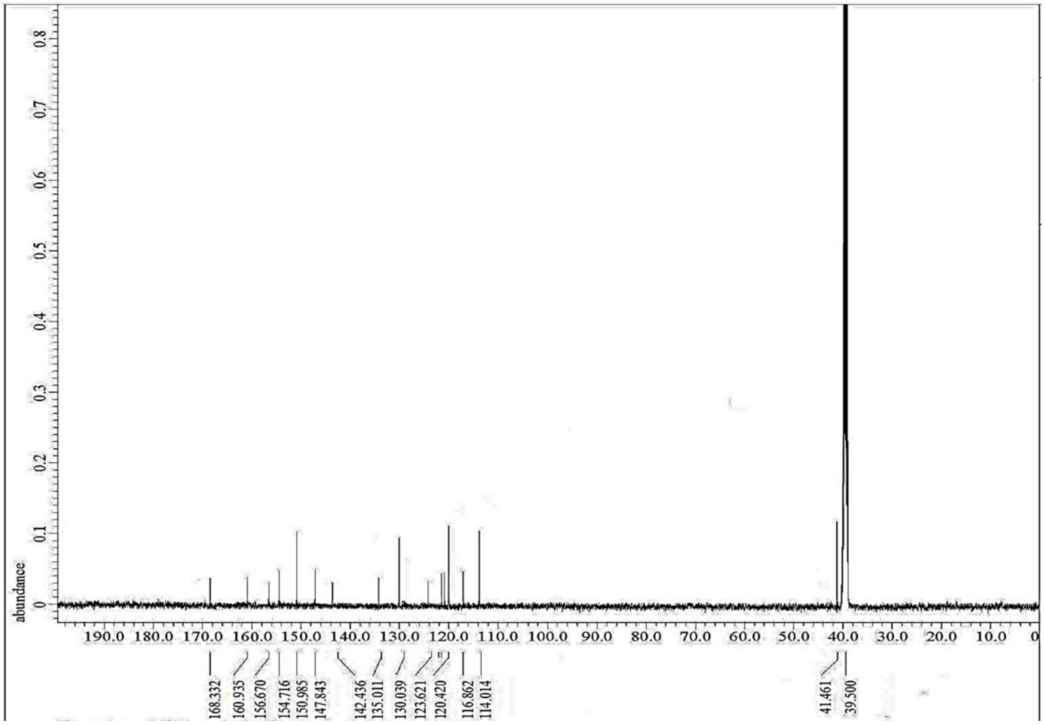

Figure 21. ^{13}C-NMR spectrum of hybrid **4a**.

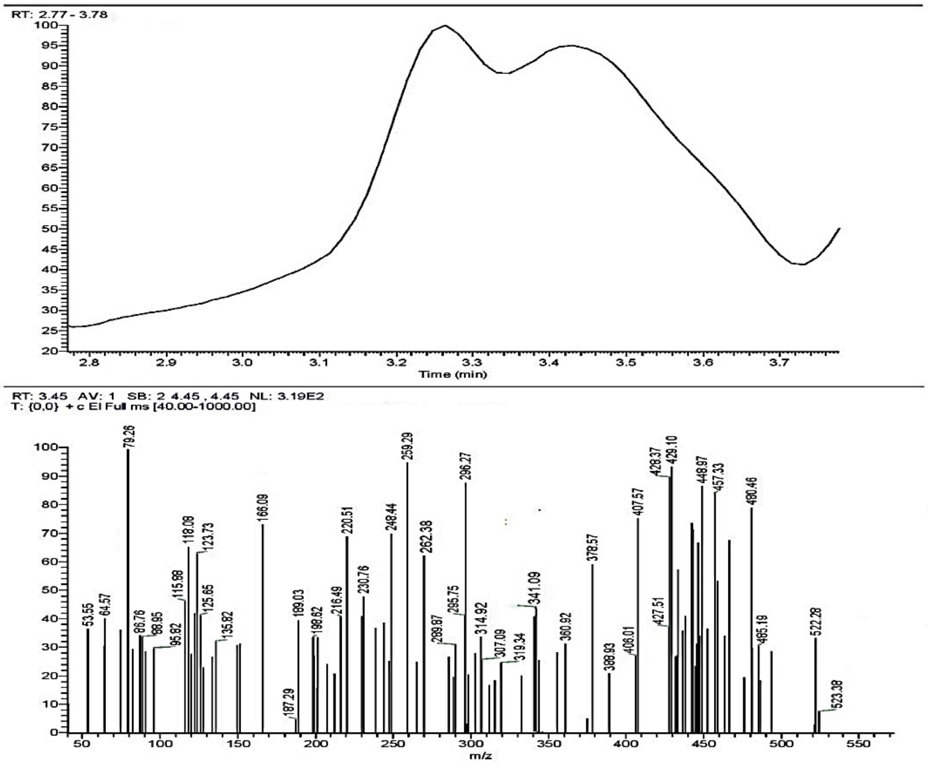

Figure S22. Mass analysis of hybrid **4a**.

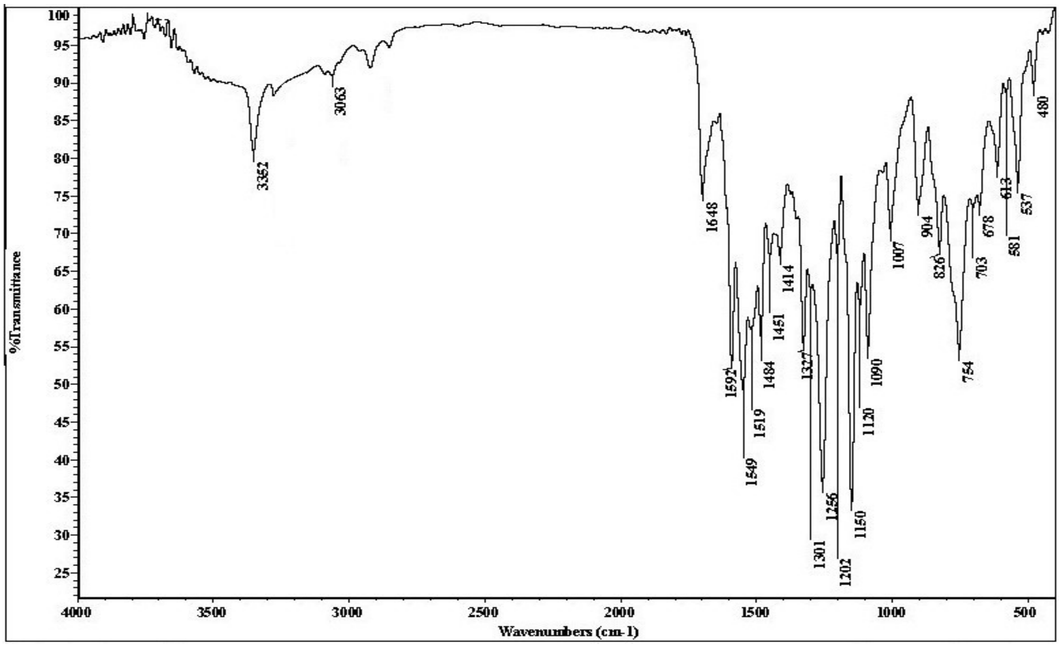

Figure S23. I.R spectrum of hybrid **4b**.

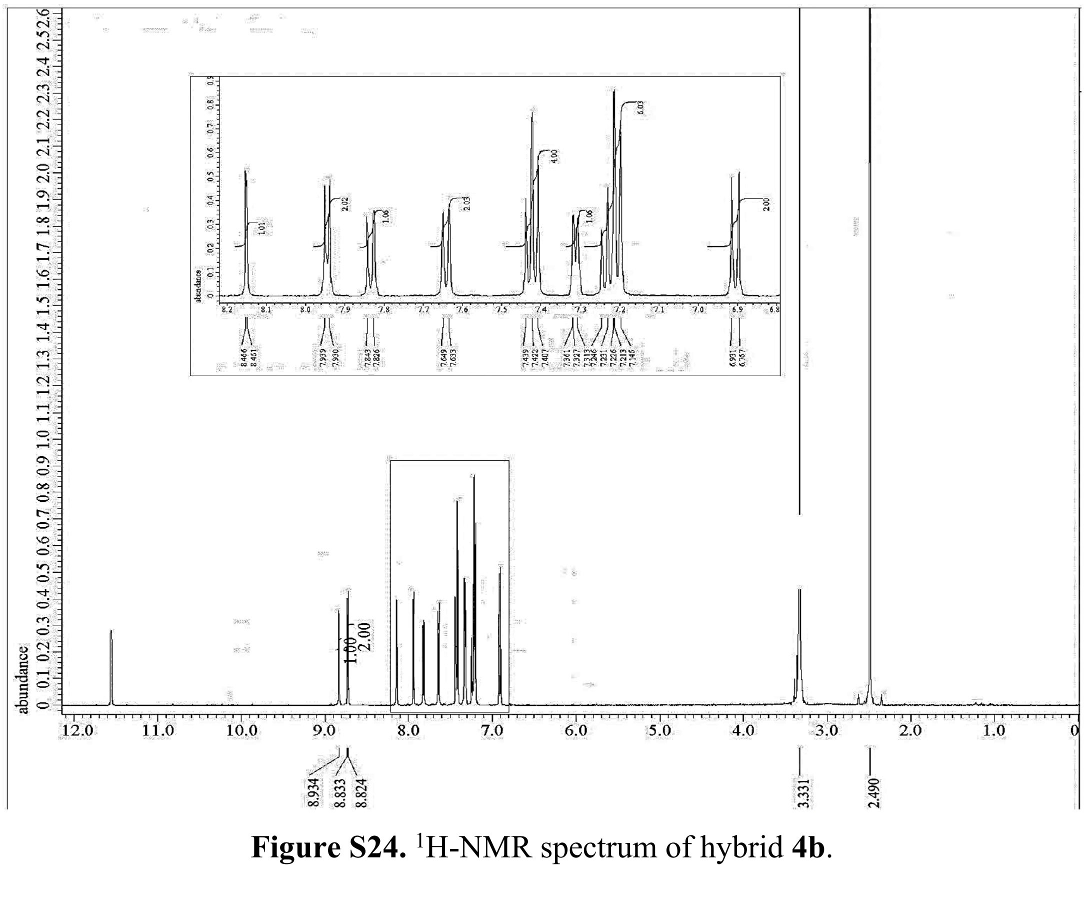

Figure S24. ^{1}H-NMR spectrum of hybrid **4b**.

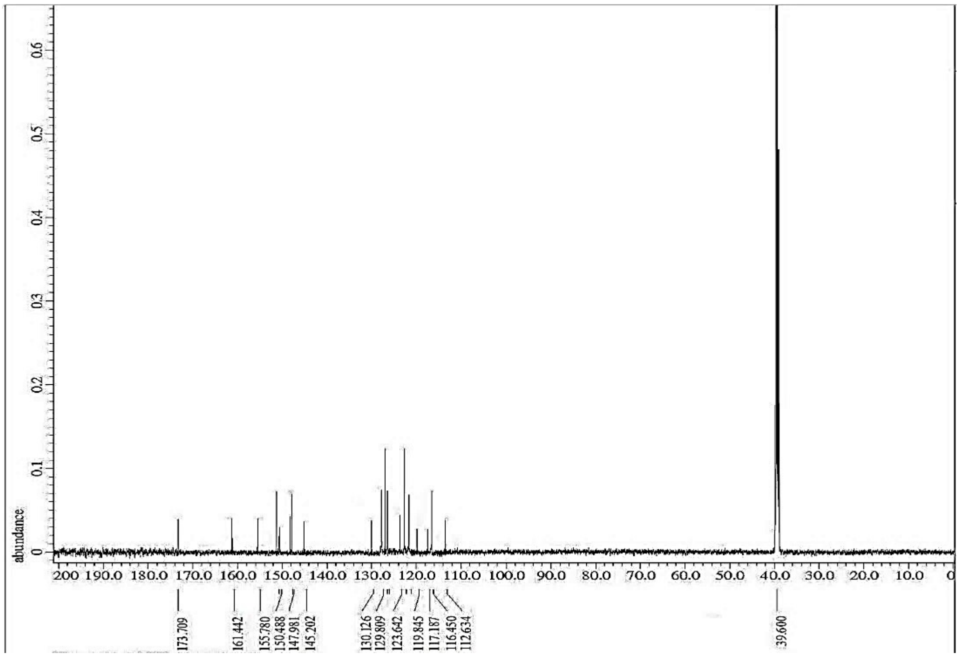

Figure S25. ^{13}C-NMR spectrum of hybrid **4b**.

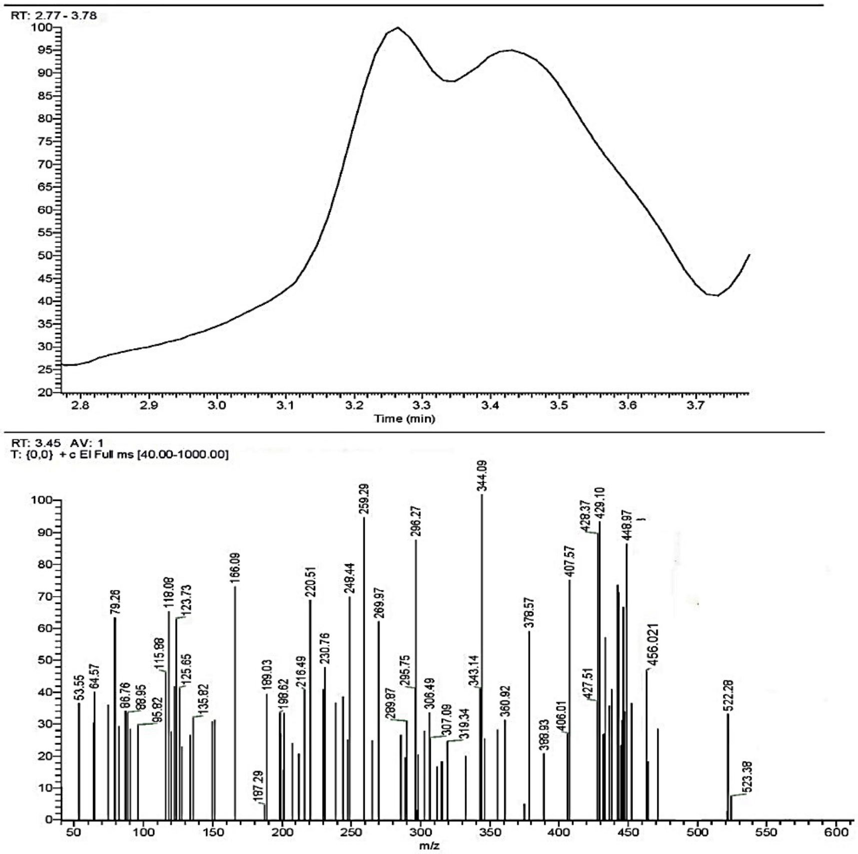

Figure S26. Mass analysis of hybrid **4b**.
